# Emergence of OXA-48-like producing *Citrobacter* species, Germany, 2011 to 2022

**DOI:** 10.2807/1560-7917.ES.2024.29.15.2300528

**Published:** 2024-04-11

**Authors:** Julian Sommer, Hannah Reiter, Janko Sattler, Elisabetta Cacace, Jessica Eisfeld, Sören Gatermann, Axel Hamprecht, Stephan Göttig

**Affiliations:** 1 Goethe University Frankfurt, University Hospital, Institute of Medical Microbiology and Infection Control, Frankfurt am Main, Germany; 2 University Hospital Cologne and Faculty of Medicine, University of Cologne, Institute for Medical Microbiology, Immunology and Hygiene, Cologne, Germany; 3 Institute of Microbiology, Department of Biology, ETH Zurich, Zurich, Switzerland; 4 German National Reference Centre for Multidrug-resistant Gram-negative Bacteria, Department of Medical Microbiology, Ruhr-University Bochum, Bochum, Germany; 5 University of Oldenburg and Klinikum Oldenburg, Institute for Medical Microbiology and Virology, Oldenburg, Germany

**Keywords:** Enterobacterales, Antimicrobial susceptibility, Carbapenemases, OXA-48, Citrobacter, Germany

## Abstract

**Background:**

Carbapenemase-producing Enterobacterales are a public health threat worldwide and OXA-48 is the most prevalent carbapenemase in Germany and western Europe. However, the molecular epidemiology of OXA-48 in species other than *Escherichia coli* and *Klebsiella pneumoniae *remains poorly understood.

**Aim:**

To analyse the molecular epidemiology of OXA-48 and OXA-48-like carbapenemases in *Citrobacter* species (spp.) in Germany between 2011 and 2022.

**Methods:**

Data of 26,822 Enterobacterales isolates sent to the National Reference Centre (NRC) for Gram-negative bacteria were evaluated. Ninety-one *Citrobacter* isolates from 40 German hospitals harbouring *bla*_OXA-48/OXA-48‑like_ were analysed by whole genome sequencing and conjugation experiments.

**Results:**

The frequency of OXA-48 in *Citrobacter freundii* (CF) has increased steadily since 2011 and is now the most prevalent carbapenemase in this species in Germany. Among 91 in-depth analysed *Citrobacter* spp. isolates, CF (n = 73) and *C. koseri* (n = 8) were the most common species and OXA-48 was the most common variant (n = 77), followed by OXA-162 (n = 11) and OXA‑181 (n = 3). Forty percent of the isolates belonged to only two sequence types (ST19 and ST22), while most other STs were singletons. The plasmids harbouring *bla*_OXA‑48_ and *bla*_OXA-162_ belonged to the plasmid types IncL (n = 85) or IncF (n = 3), and plasmids harbouring *bla*_OXA‑181_ to IncX3 (n = 3). Three IncL plasmid clusters (57/85 IncL plasmids) were identified, which were highly transferable in contrast to sporadic plasmids.

**Conclusion:**

In CF in Germany, OXA-48 is the predominant carbapenemase. Dissemination is likely due to distinct highly transmissible plasmids harbouring *bla*_OXA‑48_ or *bla*_OXA-48-like_ and the spread of the high-risk clonal lineages ST19 and ST22.

**Key public health message**

**What did you want to address in this study and why?**

Pathogenic bacteria, which are resistant against most antimicrobials, are spreading in Europe causing difficult-to-treat infections. The bacterial enzyme OXA-48 mediates resistance against carbapenems, a group of last-resort antimicrobials and has been recently found in the bacterium *Citrobacter freundii*. We therefore investigated the emergence of these bacteria in Germany in a multicentre study over a period of 12 years.

**What have we learnt from this study?**

*Citrobacter freundii* with the resistance factor OXA-48 are increasingly found in patients treated in hospitals throughout Germany. We identified two parallel modes of spreading of these multidrug-resistant bacteria. Firstly, the nationwide distribution of the two highly prevalent bacterial lineages of sequence type (ST) ST19 and ST22. Secondly, unique genetic features enabling the efficient transfer of the OXA-48 gene between different bacteria.

**What are the implications of your findings for public health?**

*Citrobacter freundii* contributes to the spread of multidrug-resistant bacteria and the antimicrobial resistance factor OXA-48. Future multi-country surveillance programmes should closely monitor the increasing spread of OXA-48 producing *Citrobacter* to limit the worldwide dissemination of bacterial multidrug resistance.

## Introduction

Carbapenem-resistant Enterobacterales are a global public health threat [1]. Resistance to carbapenems in Enterobacterales is primarily caused by carbapenemases, bacterial enzymes that hydrolyse carbapenems and most other beta-lactam antimicrobials. Other mechanisms include hyperproduction of ampicillinase (Amp)C and/or extended spectrum beta-lactamases (ESBL) in combination with functional modifications of porins [2]. The spread of carbapenemases has been largely attributed to epidemiologically successful clonal lineages (e.g. *Klebsiella pneumoniae* sequence type (ST)258 and *Escherichia coli* ST410) and horizontal gene transfer (HGT) of plasmids encoding carbapenemase genes [3,4].

The carbapenemase oxacillinase (OXA)-48 and closely related variants, commonly referred to as OXA-48-like (e.g., OXA-181, OXA-162, OXA-484), are the most prevalent carbapenemases in Enterobacterales in western Europe, North Africa and the Middle East [5]. The carbapenemase type OXA-48 is encoded by *bla*_OXA-48_, which is frequently located on a transferable 63 kilobase (kb) IncL plasmid, albeit a chromosomal localisation has also been reported in *E. coli* and rarely in *K. pneumoniae* [5-7]. Other OXA-48 variants are found on different plasmids or are chromosomally encoded [8-10]. The main driver of OXA-48 and OXA-48-like carbapenemases dissemination is considered the HGT of *bla*_OXA-48-like_-harbouring plasmids, which can occur at high frequencies within and between bacterial species [5]. While OXA-48 and OXA-48-like carbapenemases have been primarily described in *E. coli* and *K. pneumoniae* [10], they have also been reported in other facultative pathogenic Enterobacterales species, including *Enterobacter cloacae*, *Serratia marcescens,* *Proteus mirabilis* and *Citrobacter* *freundii* [11-14]. The role of these species in the dissemination of these carbapenemases is still unclear.

*Citrobacter* species (spp.) are increasingly recognised as clinically relevant pathogens, causing both nosocomial and community-acquired infections [15]. Recent evidence suggests that the rate of infections caused by carbapenemase-producing *Citrobacter* spp. is increasing [13,16]. Yet, there is limited information on the molecular epidemiology of OXA-48- and OXA-48-like producing *Citrobacter* spp., including its genetic diversity and genetic support of *bla*_OXA-48_ and *bla*_OXA-48-like_.

Here, we describe the molecular epidemiology of clinical OXA-48 and OXA-48-like-producing *Citrobacter* spp. isolates, obtained from surveillance programmes in Germany over a period of 12 years (2011–2022). We provide an in-depth assessment of genetic relatedness, resistome and mobilome and unique genetic features of OXA-48 and OXA-48-like harbouring *Citrobacter* spp. from Germany to obtain a more comprehensive understanding of their emergence.

## Methods

### Bacterial isolates and antimicrobial susceptibility testing

For epidemiological analysis, data from Enterobacterales isolates sent to the German National Reference Centre for Multidrug-resistant Gram-negative Bacteria (NRC) for detection of carbapenem resistance mechanisms between January 2011 and December 2022 were analysed. The sampling and testing procedure has been previously described and is summarised in the Supplementary methods [17,18]. Of all isolates, a subset of 90 *Citrobacter* spp. isolates harbouring *bla*_OXA-48_/*bla*_OXA-48-like_ collected between January 2011 and December 2022 and one additional *C. freundii* isolate, collected in 2009, were chosen for further analysis. These isolates were from 40 different German hospitals and selected based on geographical distribution to obtain a representative dataset on OXA-48 producing *Citrobacter* from Germany. These isolates were analysed by whole genome sequencing, conjugation studies and susceptibility testing. Antimicrobial susceptibility testing was performed with antibiotic gradient strips (bioMerieux, Marcy-l'Étoile, France or Liofilchem, Roseto degli Abruzzi, Italy), except for fosfomycin and colistin, which were tested by agar dilution (Liofilchem) and broth microdilution (Merlin, Bornheim, Germany), respectively. Susceptibility testing and interpretation of minimal inhibitory concentrations (MIC) were performed according to EUCAST guidelines v13.0 (https://www.eucast.org/clinical_breakpoints). Tigecycline MIC was interpreted according to breakpoints established for *E. coli* and *Citrobacter koseri*.

### Whole genome sequencing

Whole genome sequencing was carried out for all 91 *Citrobacter* isolates using short-read (MiSeq or NovaSeq platform, Illumina, San Diego, the United States (US)) and long‑read sequencing technology (MinION platform, Oxford Nanopore Technologies, Oxford, the United Kingdom (UK)) as described previously [19] and in the Supplementary methods.

### Sequencing data processing

Sequencing data processing was performed as previously described [5]. Briefly, raw data was trimmed and hybrid de novo assembly was conducted using Unicycler v0.4.8 [20]. Assemblies were annotated employing Prokka v1.14.6 using the plasmid pKOI-34 (National Center for Biotechnology Information (NCBI) accession number AB715422.2) as reference [21]. Additional annotation was performed with ABRicate v1.0.1 (https://github.com/tseemann/abricate) including different databases, as described in the Supplementary methods.

### Phylogenetic and plasmid sequence analysis

For phylogenetic analysis of *Citrobacter* spp. isolates, core genomes were calculated using the software panaroo v1.2.9 [22]. Core genome single nucleotide polymorphisms (SNPs) were used for maximum likelihood phylogenetic tree construction with IQ-TREE 2 v2.2.0 (http://www.iqtree.org/) and the best fitting substitution model as identified by ModelFinder [23]. As no consensus SNP cut-off to define clonal isolates of *Citrobacter* spp. is available, we used an approximated mutation rate combined with epidemiological data and antimicrobial resistance markers to define a SNP cut-off of 5 as described in [12] and the Supplementary methods. Plasmid sequences were compared using a hash-based algorithm, implemented in Mashtree v1.2 (https://github.com/lskatz/mashtree) with a kmer length of 21 and a sketch size of 10,000, constructing a phylogeny using the integrated neighbour-joining model [24].

Publicly available sequences of *Citrobacter* spp. isolates were downloaded from the NCBI Pathogen detection database (https://www.ncbi.nlm.nih.gov/pathogens/isolates/) and filtered as described in the Supplementary methods, resulting in a dataset of 1,946 assemblies. The multilocus sequence typing (MLST) allele matrix of these assemblies was parsed to the Grapetree software (https://github.com/achtman-lab/GrapeTree) for construction of a MLST-based phylogeny using the MSTree V2 algorithm [25].

### Horizontal gene transfer of *bla*_OXA-48-like_ harbouring plasmids

Conjugation of plasmids harbouring *bla*_OXA-48_ or *bla*_OXA‑48-like_ genes was conducted by liquid mating as previously described [5,26]. Briefly, a mixture of *bla*_OXA‑48-like_ harbouring *Citrobacter* spp. donors and the sodium azide-resistant *E. coli* J53 recipient were incubated in brain heart infusion broth (Becton Dickinson, Franklin Lakes, US) for 16 h at 37°C. Subsequently, cell suspensions were streaked on chromogenic agar plates (Carl Roth, Karlsruhe, Germany) containing 100 mg/L sodium azide and 15 mg/L amoxicillin-clavulanate to select for transconjugants (Tc). The Tc were tested for the presence of *bla*_OXA-48-like_ genes by disk diffusion and PCR [27]. Frequency of conjugation was determined by dividing the number of Tc by the number of recipient colonies [5].

## Results

### Emergence of OXA-48 producing *Citrobacter freundii* in Germany

**Figure 1:**
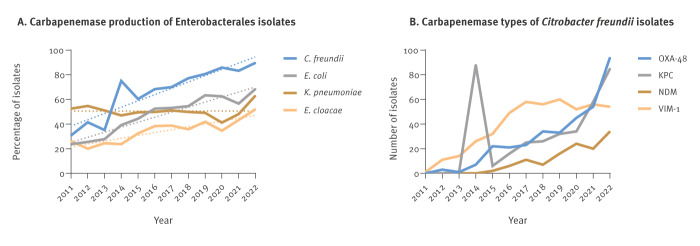
Carbapenemase production in Enterobacterales isolates (n = 26,822) and carbapenemase types in *Citrobacter freundii* isolates (n = 91) sent to the German National Reference Centre for Multidrug-resistant Gram-negative Bacteria, Germany, 2011–2022

The percentage of Enterobacterales isolates (n = 26,822), sent to NRC between 2011 and 2022, which were carbapenemase positive has increased in the four most prevalent species (*K. pneumoniae, E. coli, C. freundii, E. cloacae*) (Figure 1A). Additional data on prevalence of carbapenemase-producing Enterobacterales are shown in the Supplementary Figure S1A. Since 2014, species of the *C. freundii* complex had the highest rate of carbapenemase production. The four most prevalent carbapenemases in *C. freundii* were OXA-48, *K. pneumoniae* carbapenemase (KPC), New Delhi metallo-beta-lactamase (NDM) and Verona integron‒encoded metallo-beta-lactamase (VIM)-1 (Figure 1B). Until 2020, VIM‑1 was the most frequent carbapenemase, except for a KPC peak in 2014 due to a large regional outbreak [28]. Since 2021, the most prevalent carbapenemase in *C. freundii* is OXA‑48. Moreover, the frequency of *bla*_OXA-48_ in *C. freundii* has increased steadily since 2011 and is higher than in any other species, as can be seen in the Supplementary Figure S1B.

*C. freundii*: *Citrobacter freundii*; *E. cloacae*: *Enterobacter cloacae*; *E. coli*: *Escherichia coli*; *K. pneumoniae*: *Klebsiella pneumoniae*; KPC: *Klebsiella pneumoniae* carbapenemase; NDM: New Delhi metallo-beta-lactamase; OXA-48: oxacillinase-48; VIM-1: Verona integron‒encoded metallo-beta-lactamase.

Panel A. Carbapenemase positivity of the four most prevalent Enterobacterales species with dotted trendlines.

Panel B. Absolute numbers of *Citrobacter freundii* isolates stratified by carbapenemase.

### Epidemiology of OXA-48-like producing *Citrobacter* species in Germany

**Figure 2:**
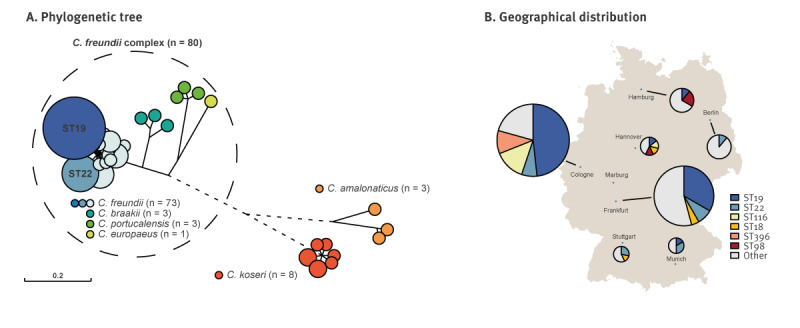
Emergence of OXA-48 and OXA-48-like producing isolates of *Citrobacter* species, Germany, 2011–2022 (n = 91)

Whole genome sequencing of all isolates was performed to generate hybrid assemblies and enable in-depth genetic analyses. The genetic characteristics are displayed in the Supplementary Table S1. Isolates belonged to six species: *C. koseri* (n = 8) and *C. amalonaticus* (n = 3) and four *C. freundii* complex species of *C. freundii* (n = 73), *C. braakii* (n = 3), *C. portucalensis* (n = 3) and *C. europaeus* (n = 1) (Figure 2A). A diverse set of 45 different STs was revealed by MLST. The two most prevalent STs were ST19 (n = 25) and ST22 (n = 9) (Figure 2B). Most of the ST19 isolates (n = 23) were derived from densely populated areas in western and central Germany (Figure 2B). Less frequently observed STs were ST116 (n = 5), ST18 (n = 3), ST396 (n = 3) and ST98 (n = 3), whereas the other STs were mostly singletons. The ST116 isolates were only detected in central or western Germany and ST98 isolates only in northern Germany. The isolates were obtained from a diverse range of clinical specimens, most frequently from rectal swabs (n = 38), urine samples (n = 20) and skin (n = 10). Twenty-three isolates were derived from intraoperative tissue samples (n = 10), deeper respiratory tract specimens (n = 6), blood cultures (n = 4) or wounds (n = 3), suggesting potential *Citrobacter*-sustained infections.

*C*.: *Citrobacter*; OXA: oxacillinase; ST: sequence type.

Panel A. Unrooted phylogenetic tree of clinical isolates analysed in this study and constructed using 2,798 core genes. Node size is proportional to the number of isolates belonging to each node.

Panel B. Geographical distribution of OXA-48/OXA-48-like harbouring *Citrobacter* spp. isolates (n = 91) in different German regions. Pie chart size is proportional to the numbers of isolates at each site. Number of isolates per region: Berlin n = 9; Cologne/Ruhr n = 30; Frankfurt/Rhine-Main n = 24; Hamburg n = 9; Hannover n = 7; Munich n = 6; Stuttgart n = 6.

### Antimicrobial susceptibility of OXA-48 and OXA-48-like producing *Citrobacter* species

**Figure 3:**
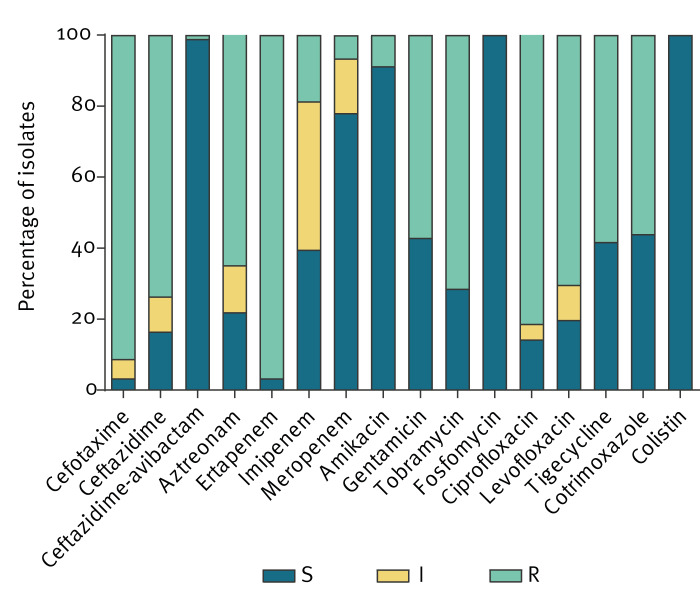
Antimicrobial susceptibility of OXA-48 and OXA-48-like producing *Citrobacter* species isolates, Germany, 2011–2022 (n = 91)

Susceptibility of isolates to carbapenems varied from 3% (n = 3) for ertapenem to 81% (n = 74) for imipenem and 93% (n = 85) for meropenem (Figure 3). Only five isolates exhibited MICs at or below the EUCAST screening breakpoints for suspected carbapenemase production, which can be seen in the Supplementary Figure S2. Isolates were frequently resistant to cefotaxime (83/91; 91%) and ceftazidime (67/91; 74%), indicating co-expression of ESBLs and/or hyperproduction of AmpC enzymes. The lowest resistance rates were recorded for colistin (0%), fosfomycin (0%), ceftazidime-avibactam (1/91; 1%) and amikacin (8/91; 9%). For all other tested antimicrobials resistance rates > 50% were recorded (Figure 3).

I: susceptible, increased exposure; R: resistant; S: susceptible.

### Analysis of whole genome sequences and molecular epidemiology

**Figure 4:**
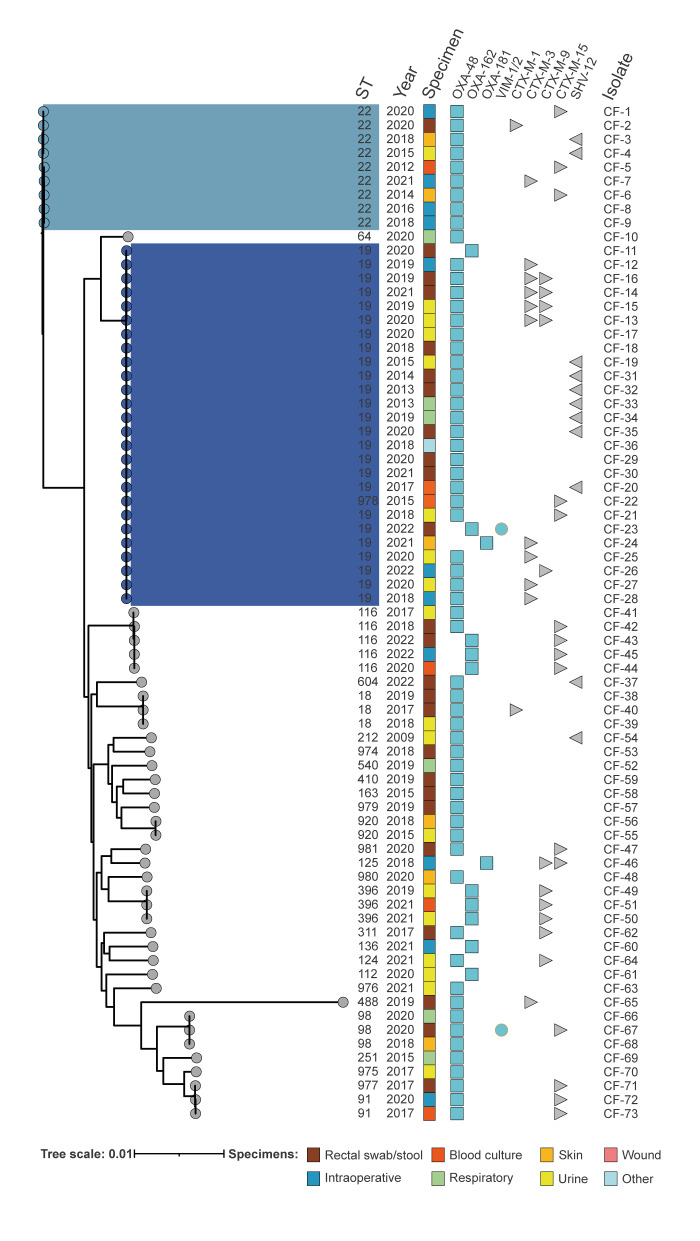
Phylogeny of OXA-48 and OXA-48-like harbouring *Citrobacter freundii*, Germany, 2011–2022 (n = 73)

CTX: cefotaximase; OXA: oxacillinase; SHV: sulfhydryl variable; ST: sequence type; VIM: Verona integron‒encoded metallo-beta-lactamase.

Core genome analysis of all 73 *C. freundii* isolates revealed two larger clusters comprising ST19/ST978 (n = 26) and ST22 (n = 9) isolates (Figure 4). The nine isolates of the ST22 cluster solely carried *bla*_OXA‑48_ and frequently co-expressed cefotaximase (CTX)-M (7/9). Isolates of the ST19/ST978 cluster encoded OXA-48 (23/26) or OXA-48-like carbapenemases (OXA‑162: 2/26; OXA-181: 1/26) and frequently co-expressed CTX-M (12/26). In contrast, isolates of the other STs (n = 38) more frequently encoded for OXA‑48-like (9/38) and less often co-expressed CTX-M enzymes (19/38). Core genome analysis of the 18 non-*C. freundii* species revealed 16 different STs with only ST854 and ST862 of the *C. koseri* species identified in two isolates, each. The proportion of co-expression of additional beta-lactamases of the CTX-M or SHV-group was lower (6/18) compared with the *C. freundii* isolates, as presented in the Supplementary Figure S3.

For each isolate, the STs, year of isolation, specimen and presence of beta-lactamase-encoding genes are shown. The carbapenemases OXA-48, OXA-162 and OXA-181 are shown by squares and VIM-1/2 by circles in light blue; beta-lactamases of the CTX-M, SHV and OXA-10-group are shown in light grey triangles. Each isolate in the tree harbours a *bla*_CMY_ gene (not shown). Isolates with the two most frequent STs (ST22 and ST 19/978) are highlighted in light blue and dark blue, respectively. The scale bar indicates the numbers of substitutions per site.

Among all 91 *Citrobacter* spp. isolates, OXA-48 (n = 77) was the most frequent enzyme, followed by OXA-162 (n = 11) and OXA-181 (n = 3). Two isolates co-expressed VIM-1 or VIM‑2, respectively. One OXA-48 isolate additionally produced OXA-917, a yet uncharacterised enzyme of the OXA-427-like carbapenemase family [29]. All isolates collected until 2017 encoded *bla*_OXA-48_, while OXA-162 and OXA-181 variants emerged in 2018.

To identify possible clonal isolates, a core genome phylogeny was constructed and the number of SNPs between isolates was analysed. The core genome SNPs between isolates of all *Citrobacter* species ranged from 0 to 419,862, resulting in the segregation of the *Citrobacter freundii* complex species and the two distinct species *C. koseri* and *C. amalonaticus* (Figure 2A). Using a cut-off of 5 SNP per year, four small clusters of three or more potentially clonal *C. freundii* isolates were identified and matched with epidemiological data, as seen in the Supplementary Figure S4. The largest cluster of five isolates originated from the Rhine-Main region (isolates CF-31, CF‑32, CF-33, CF-35 and CF-36). These isolates were collected between 2013 and 2020 with a maximum SNP difference of 12, suggesting a common origin. Three unrelated smaller clusters of three isolates each were identified in the Cologne region 2019–2021. These clusters differed in the presence of OXA‑48‑like variants and/or OXA-48-like harbouring plasmids.

### Genetic support of OXA-48 and OXA-48-like carbapenemases

**Figure 5:**
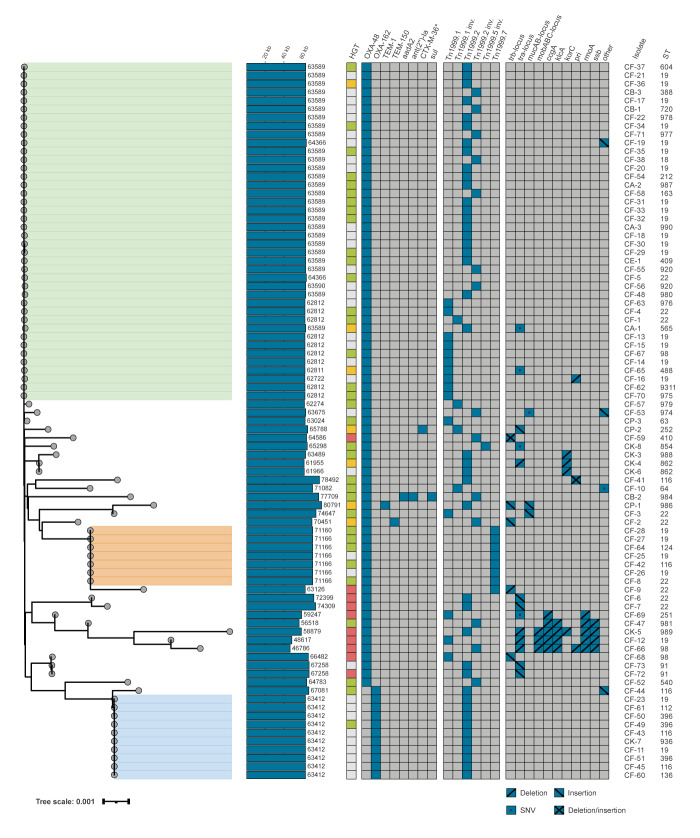
Phylogeny of IncL OXA-48 and OXA-48-like plasmids from isolates of *Citrobacter* species, Germany, 2011-2022 (n = 85)

CA: *Citrobacter amalonaticus*; CB: *Citrobacter braakii*; CE: *Citrobacter europaeus*; CF: *Citrobacter freundii*; CK: *Citrobacter koseri*; CP: *Citrobacter portucalensis*; CTX: cefotaximase; HGT: horizontal gene transfer; kb: kilobase; OXA: oxacillinase; SHV: sulfhydryl variable; ST: sequence type; TEM: Temoniera; Tn: transposon.

We determined the genetic support and mobilome for all isolates (Figure 5). In all isolates, *bla*_OXA-48_/*bla*_OXA-48-like_ genes were located on plasmids (as determined by circular assembled plasmids). Of these, 85/91 plasmids belonged to the incompatibility group IncL with sizes ranging from 46,786 bp to 80,791 bp. Three plasmids belonged to the IncX3 group (all OXA‑181) and three plasmids to the IncF group (all OXA‑48), presented in the Supplementary Figure S5. Interestingly, *C. portucalensis* isolate CP-2 encoded two copies of *bla*_OXA‑48_, one on a 65.8 kb size IncL plasmid and one located on the chromosome, disrupting the gene *yjjW*, a putative oxidoreductase. In both locations, *bla*_OXA-48_ was flanked by IS*1999* insertion sequences.

For each plasmid, its length in base pairs, semiquantitative conjugation frequency, presence of antimicrobial resistance genes, Tn*1999* transposon variant, mutations, harbouring isolate and ST are shown. Genes with < 100% nucleotide identity are marked with an asterisk. The column ‘Other’ includes three hypothetical proteins: N8-B: WP_004206908.1, N25: WP_023302502.1 and D2054-3: WP_015062822.1. Colours in the HGT column represent HGT frequency: green: 10^-4^-10^-1^; yellow: 10^-6^-10^-5^; red: no HGT; white: not determined. The scale bar indicates the number of substitutions per site.

Sequence analysis of the 85 IncL plasmids revealed three larger clusters with sizes of approximately 63 kb (n = 40), 63.4 kb (n = 10) and 71.2 kb (n = 7), representing 57/85 isolates (Figure 5). The largest cluster comprised 40 plasmids from isolates of the *C. freundii* complex or *C. amalonaticus*, ranging from 62.7 to 64.4 kb*.* These plasmids were highly similar to the previously described epidemic 63 kb-IncL-plasmid harbouring *bla*_OXA-48_ with a sequence identity of > 99.9% (when excluding the Tn*1999* sequence) [5,8]. The second cluster consisted of six identical plasmids from *C. freundii* isolates with a size of 71,166 bp and one plasmid of 71,160 bp, harbouring the Tn*1999.7* transposon [12]. The third cluster included ten identical plasmids of 63,412 bp from *C. freundii* and *C. koseri* isolates, harbouring *bla*_OXA‑162_ within Tn*1999.2* [30] (Figure 5). The remaining 28 unclustered plasmids were more diverse, differing in size (46,786 to 80,791 bp), Tn*1999* transposon variants and carriage of additional resistance genes or various mutations (Figure 5). In contrast to the other species, *C. koseri* only harboured either sporadic plasmids or non-IncL plasmids (with exception of isolate CK‑7).

### Transmission of OXA-48 and OXA-48-like plasmids from isolates of *Citrobacter* species

To assess the potential of the *bla*_OXA-48_ and *bla*_OXA-48-like_-carrying plasmids for dissemination, we systematically tested their transfer capabilities by conjugation. The 57 plasmids from the three clusters comprise 67% of all 85 identified IncL plasmids. Of these, 26 representative plasmids from all clusters were selected (based on e.g. Tn*1999* variant, OXA-48-like variant or sequence length) for conjugation studies with *E. coli* J53 serving as the recipient strain [5]. All tested plasmids were successfully transferred, 23 with high mean frequencies (3.5 × 10^-4^ to 4.1 × 10^‑1^) and three plasmids with lower mean frequencies (1.8 × 10^‑6^ to 9.5 × 10^-5^) (Figure 5) and the Supplementary Figure S6. Detailed sequence analysis of the genes essential for HGT and plasmid stabilisation revealed SNPs in *traN* and *traU* in two of the plasmids with reduced conjugation frequencies.

In contrast, when mobilisation of the unclustered IncL plasmids was analysed, 10 of the 25 plasmids could not be conjugated. Sequence analysis revealed SNPs, deletions or insertions in genes associated with plasmid mobilisation and/or stability (Figure 5). Insertions, deletions or SNPs in genes of the *trb* locus (*trbA*, *trbB* and/or *trbC*; plasmid transfer) or the *tra* locus (*traH*, *traI*, *traJ*, *traK*, *traU*, *traW* and/or *traY*; conjugative transfer protein) impaired or abolished plasmid mobilisation. In contrast, modifications of the *muc* locus (transcriptional repressor), *mob* locus (plasmid mobilisation) or of the genes *ccgA*, *klcA*, *korC*, *pri*, *rmoA* and *ssb* did not prevent mobilisation (Figure 5).

### Worldwide clonal lineages of carbapenemase producing isolates of *Citrobacter* species

**Figure 6:**
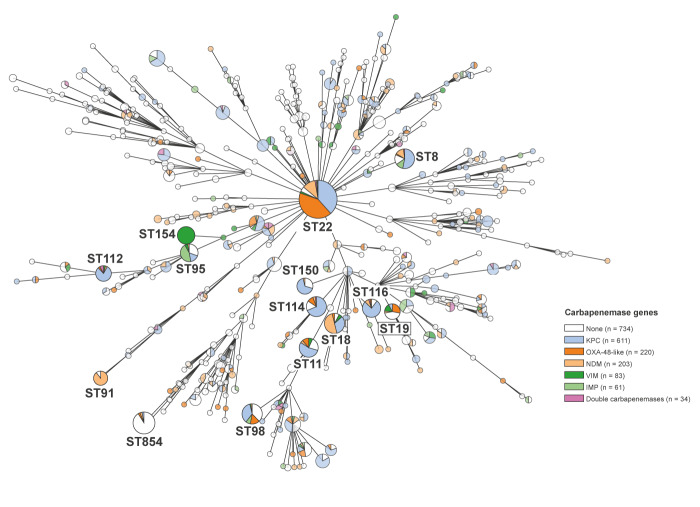
Multilocus sequence typing-based phylogeny of isolates of *Citrobacter* species available from public sequence databases with and without carbapenemase genes, 2003-2022^a^ (n = 1,946)

IMP: imipenemase; KPC: *Klebsiella pneumoniae* carbapenemase; NDM: New Delhi metallo-beta-lactamase; OXA-48: oxacillinase-48; ST: sequence type; VIM: Verona integron‒encoded metallo-beta-lactamase.

^a^ Ten of the 1,946 isolates were recovered between 1937 and 2002.

To compare the *Citrobacter* isolates from this study with international isolates, we generated an ST-based phylogeny based on 1,946 *Citrobacter* spp. assemblies from the NCBI database and systematically assessed the presence of carbapenemase genes *bla*_OXA‑48_/*bla*_OXA‑48-like_, *bla*_NDM_, *bla*_KPC_, *bla*_IMP_ and *bla*_VIM_ (Figure 6). Among these, ST22 was by far the most abundant ST (n = 322) and frequently associated with carbapenemases (308/322, 96% carbapenemase-positive), followed by ST854 (n = 65), ST98 (n = 53) and ST114 (n = 50), presented in the Supplementary Figure S7. Thirteen of the 14 most abundant STs (except ST854) harboured carbapenemase genes (> 50% sequences/ST) with KPC (n = 611) being the most frequent, followed by OXA‑48/OXA-48-like (n = 220) and NDM (n = 203). Carbapenemase genes were detected in 128 different STs, but certain associations were apparent: KPC (STs 8, 11, 112, 114, 116, 150), NDM (ST18, ST91), imipenemase (IMP)/ST95 and VIM/ST154. Notably, *bla*_OXA-48_ and *bla*_OXA-48-like_ were the most prevalent carbapenemase genes in ST19 and ST22, which were also the two most prevalent STs in this study. Isolates of ST22 producing OXA-48-like carbapenemases were mainly identified in North America, western Europe and Southeast Asia, whereas ST19 isolates were only found in Germany and France, presented in the Supplementary Figure S7 and S8. Phylogenetic comparison of the international isolate collection with the identified clustered isolates from Germany using the proposed SNP cut-off revealed no closely related isolates from other countries.

Nodes represent sequence types (ST) with diameters proportional to the number of the corresponding assemblies. Presence and proportion of carbapenemase genes are indicated by colours. Only STs comprising at least 25 isolates are shown.

## Discussion

Oxacillinase OXA-48 and OXA-48-like carbapenemases are the most common carbapenemases in Enterobacterales in Europe [5]. While these carbapenemases are commonly identified in *E. coli* and *K. pneumoniae*, they are being increasingly detected in other Enterobacterales species [31]. Analysis of the isolates submitted to the German NRC between 2011 and 2022 showed an increase in the detection of OXA-48 and OXA-48-like carbapenemases in general and specifically of OXA-48-producing *C. freundii*. Interestingly, carbapenem-resistant *C. freundii* isolates are more likely to carry carbapenemases than other carbapenem-resistant Enterobacterales species, indicating the emerging role of *Citrobacter* spp. in the dissemination of carbapenemases.

The 91 *Citrobacter* spp. isolates analysed here were collected over a period of more than 10 years from different regions in Germany. The most common STs were ST19 and ST22, highly prevalent in central Germany, but also found in other regions. Surveillance programmes from Germany and France have also identified ST19 and ST22 *C. freundii* isolates carrying OXA-48 and OXA-48-like carbapenemases, indicating the importance of these dominant clonal lineages for the spread of OXA-48 [11,13]. Whereas ST19 seems prevalent in Germany and Europe, ST22 is the most prevalent lineage worldwide and can be considered as a high-risk clonal lineage [13,31].

Carbapenemase types OXA-48 and OXA-48-like carbapenemases were detected in six different *Citrobacter* species, with *C. freundii* being the most abundant (80%). Other studies of nosocomial outbreaks including *Citrobacter* spp. isolates carrying OXA-48 carbapenemases showed similar species distributions [11]. More recently, *C. portucalensis* isolates carrying VIM and OXA-48 carbapenemases have been described [32]. These studies and our data indicate that the underlying molecular epidemiology and OXA-48 plasmid architecture differs between *C. freundii* and *C. portucalensis*. However, *C. portucalensis* is commonly misidentified as *C. freundii* by Matrix-assisted laser desorption/ionization (MALDI-TOF) mass spectrometry and is likely underreported for this reason [33]. In one *C. portucalensis* isolate, we identified a chromosomal copy of *bla*_OXA-48_ in addition to an IncL-localised copy. This is concerning, since *bla*_OXA-48_ is thereby stably integrated into the genome and can be transmitted both horizontally and vertically. Given the increase of *Citrobacter* spp. involved in the carbapenemase dissemination with distinct epidemiological pattern, future surveys should include methods that can distinguish *C. portucalensis* [32,33].

Susceptibility testing revealed a high rate of co-resistance to non-beta-lactams, with overall few antimicrobials remaining left for treatment. In case of infections, ceftazidime-avibactam, fosfomycin, colistin and amikacin could be suitable treatment options with resistance rates of < 10%. Resistance rates to the non-beta-lactam antimicrobials tobramycin, gentamicin, ciprofloxacin and tigecycline were higher compared with other studies analysing OXA-48 producing *Citrobacter* spp., suggesting acquisition of additional antibiotic resistance genes [13]. A global surveillance study analysing carbapenemase-producing *Citrobacter* spp. revealed only sporadic resistance to ciprofloxacin, gentamicin, tobramycin or tigecycline (all < 10%). In contrast, > 50% of our isolates were resistant to these drugs, indicating country-specific differences in epidemiology [32].

We observed a wide distribution of different STs, as described for other Enterobacterales carrying OXA-48 and OXA-48-like carbapenemases [5]. This polyclonality has been linked to efficient HGT of the highly transmissible IncL plasmid, rather than the dissemination of a single successful lineage [34]. However, the predominance of ST19 and ST22 observed here suggests additional modes of dissemination for *Citrobacter* spp. Detailed analysis of the ST19 isolates revealed epidemiologically distinct lineages within this group, carrying OXA-48, OXA‑162 or OXA-181. Therefore, OXA‑48 and OXA-48-like carbapenemases in this ST have probably been acquired in multiple events. The presence of several additional antibiotic resistance genes in these isolates supports this hypothesis. Recent studies described varying fitness effects of identical IncL OXA-48 plasmids on different STs in *K. pneumoniae, Klebsiella variicola*, *E. coli*, *C. freundii* and *Kluyvera ascorbata*, with positive or no impact on the fitness of epidemiologically successful STs and negative fitness effect in sporadically detected STs [35,36]. Further studies should hence analyse potential beneficial effects of the broadly disseminated OXA-48 and OXA-48-like carrying plasmids on ST19 and ST22 strain fitness.

Compared with previous studies, we employed a combined short- and long-read sequencing approach to determine the circularised plasmid sequences and flanking regions for all OXA‑48 and OXA-48-like encoding genes. In all isolates, *bla*_OXA-48_ and *bla*_OXA-48-like_ genes were identified on plasmids of the IncL (*bla*_OXA-48_, *bla*_OXA-162_), IncF (*bla*_OXA-48_) or IncX3 group (*bla*_OXA-181_). Most IncL plasmids were highly similar to the previously described epidemic IncL ~63 kb plasmid, harbouring *bla*_OXA-48_ located within Tn*1999*-like transposons [5,8]. These plasmids were identified in *Citrobacter* spp. isolates originating from hospitals all over Germany, demonstrating a similar distribution pattern between *Citrobacter* spp. and other Enterobacterales carrying OXA-48-like carbapenemases.

Our data show that these plasmids can be transferred to *E. coli*, supporting intergenera transferability as a possible reason for their rapid spread. A second cluster of IncL plasmids harbouring the recently described Tn*1999.7* transposon and a cluster of IncL plasmids encoding *bla*_OXA‑162_ were identified in western Germany, suggesting a local spread of these plasmids in a nosocomial environment. Tn*1999.7* has been associated with increased plasmid stability, even in absence of antibiotic pressure and may promote the spread of OXA-48 in the future [12]. Furthermore, we identified a group of 28 unclustered plasmids varying in sequence length and with different insertions, deletions or SNPs compared to the epidemic ~63 kb IncL plasmid. While many of these variations did not affect plasmid transmission, specifically mutations in the genes of the *tra-* and *trb* loci resulted in impaired or missing conjugation of the plasmids [37]. Accordingly, these plasmids occurred only sporadically in the analysed *Citrobacter* spp. isolates.

Our study has some limitations: the number of in-depth analysed isolates is lower compared with studies on other Gram-negative bacteria like *K. pneumoniae* or *Acinetobacter baumannii* due to the overall lower incidence of carbapenem-resistant *Citrobacter* in Germany. Furthermore, sampling sites were unevenly distributed and most isolates were collected from western and central Germany. However, our isolates originate from all parts of Germany and were collected over more than 10 years, providing a valuable insight into the spread of *Citrobacter* producing OXA-48/OXA-48-like carbapenemases*.*

Our study contributes to the better understanding of the epidemiology of OXA-48 producing *Citrobacter* spp. According to our results, it is reasonable to hypothesise that ST19 and ST22 will be the dominant clonal lineages in the future, and that the diversity of both *bla*_OXA‑48‑like_ carbapenemases and IncL plasmids (e.g., 62.8 kb IncL and Tn*1999.7* plasmids) will increase. Further studies should investigate chromosomal integration of *bla*_OXA-48_ in *C. portucalensis* (and other *Citrobacter* spp.) and their dissemination. We suggest that future international surveillance programmes should include OXA-48-like producing *Citrobacter* spp. and consider reliable identification of non-freundii *Citrobacter* species as well as MLST to closely monitor the dynamics of clonal lineages.

## Conclusion

*Citrobacter* spp. increasingly carry OXA-48 and OXA-48-like encoding carbapenemase genes on highly transmissible IncL, IncX3 and IncF plasmids. In contrast to other Enterobacterales species producing OXA-48/OXA-48-like, a high proportion of *Citrobacter* isolates (40%) belonged to only two STs (ST19 and ST22). This suggests that, highly transmissible plasmids harbouring *bla*_OXA-48_ or *bla*_OXA-48-like_ and the high-risk clonal lineages ST19 and ST22 are both responsible for the increasing dissemination of *Citrobacter* spp. with OXA-48/OXA-48-like carbapenemases.

## Supplementary Data

1443000 Supplementary Material
