# Supplementary material for: Emergence of OXA-48-like producing Citrobacter species, Germany, 2011 to 2022
Source: Euro Surveill. 2024 Apr 11;29(15):2300528. doi: 10.2807/1560-7917.ES.2024.29.15.2300528 (PMC11010590; doi:10.2807/1560-7917.ES.2024.29.15.2300528)
Supplement: Supplementary Material [file 23-00528_GOETTIG_Supplement.pdf]

Supplementary material

Emergence of OXA-48-like producing *Citrobacter* species, Germany, 2011 to 2022

This supplementary material is hosted by Eurosurveillance as supporting information alongside the article "Emergence of OXA-48-like producing *Citrobacter* spp. in Germany 2011-2022", on behalf of the authors, who remain responsible for the accuracy and appropriateness of the content. The same standards for ethics, copyright, attributions and permissions as for the article apply. Supplements are not edited by Eurosurveillance and the journal is not responsible for the maintenance of any links or email addresses provided therein.

Supplementary methods

Sampling procedure

The National Reference Centre for Multidrug-resistant Gram-negative Bacteria (NRC) in Bochum requests all primary diagnostic laboratories in Germany to voluntarily send Enterobacterales isolates that fulfil specific criteria for verification or exclusion of carbapenemase production. For *Citrobacter* sp. these criteria are elevated minimal inhibitory concentrations/decreased inhibitory zone diameters for ertapenem (>0.5 mg/L or <25 mm [10 µg]), meropenem or imipenem (< 2 mg/L or < 25 mm [10 µg]). The detailed workflow has been described previously [1]. Carbapenemase production was confirmed using a combination of phenotypic tests and carbapenemase-specific PCR amplification and amplicon sequencing [2].

Whole genome sequencing and sequencing data processing

Whole genome DNA from isolates was extracted using the DNeasy UltraClean Microbial Kit (Qiagen, Hilden, Germany). Whole genome sequencing was carried out using short-read technology utilizing a v3 reagent kit generating 150 bp paired-end reads (MiSeq or NovaSeq platform, Illumina, San Diego, USA) and additionally by long-read sequencing technology (MinION, Oxford Nanopore Technologies, Oxford, UK). Library preparation for Nanopore sequencing was done using the SQK-RBK004 rapid barcoding or the SQK-LSK109 ligation sequencing kit with the EXP-NBD104 native barcoding extension. Long-read sequencing was performed on a MinION MK1B or MK1C sequencer utilizing R9.4.1 flongle or MinION flow cells. Raw signal data was base called and demultiplexed using the super-accurate base calling model of guppy basecaller version 6.3.2. Short-reads were trimmed utilizing trimmomatic v0.39 and NanoFilt v2.8.0 for long-reads of reads, resulting in an average genome coverage of ≥100-fold for short-reads and ≥30-fold for long-reads, a N50 >100,000 bp and circulated plasmids carrying $bla_{\text{OXA-48}}$ or $bla_{\text{OXA-48}}$-like genes for all assemblies [3,4]. Bacterial species were identified utilising BIGSdb

and sequence types were identified using mlst v2.23.0 (https://github.com/tseemann/mlst) [5,6]. For annotation of plasmid incompatibility groups, antibiotic resistance genes and insertion sequences, the databases PlasmidFinder, NCBI AMRFinderPlus and ISfinder were used respectively [6–8]. Variant sites of the core genome alignments for phylogenetic analyses were extracted using snp-sites v2.5.1 [9]. Since no specific mutation rate estimation for *Citrobacter* sp. were available from literature, an approximated mutation rate derived from other Enterobacterales was used to calculate a SNP/year rate of 1.5, as previously described [10]. Starting with this cut-off, increasing thresholds of SNP were related to epidemiological data and antibiotic resistance markers, resulting in a proposed SNP cut-off of 5.0 for clonality. The use of higher SNP thresholds let to the clustering of isolates that differed in terms of epidemiological data and/or antibiotic resistance markers.

Selection of *Citrobacter* spp. from public databases for molecular epidemiology analysis

A total of 1,960 assemblies of *Citrobacter* spp. isolates were downloaded from the pathogen detection database of the National Center for Biotechnology Information (https://www.ncbi.nlm.nih.gov/pathogens/isolates/) on February 17th, 2023. Only datasets including collection date, location and an assembly were selected. Assemblies were filtered for N50 >10,000 bp and bacterial species were identified using BIGSdb [5] resulting in 1946 assemblies for further analysis. Sequence types of these assemblies were identified using mlst v2.23.0 (https://github.com/tseemann/mlst). The MLST allele matrix was used for generation of a MLST-based phylogeny utilising the MStree V2 algorithm from the Grapetree software [11]. Antibiotic resistance genes were identified using ABRicate v1.0.1 (https://github.com/tseemann/abricate) and the NCBI AMRFinderPlus database [7]. Geographical distribution of the isolates with information of country of collection was visualised using the web application Microreact version 251 (www.microreact.org) [12].

Supplementary figures

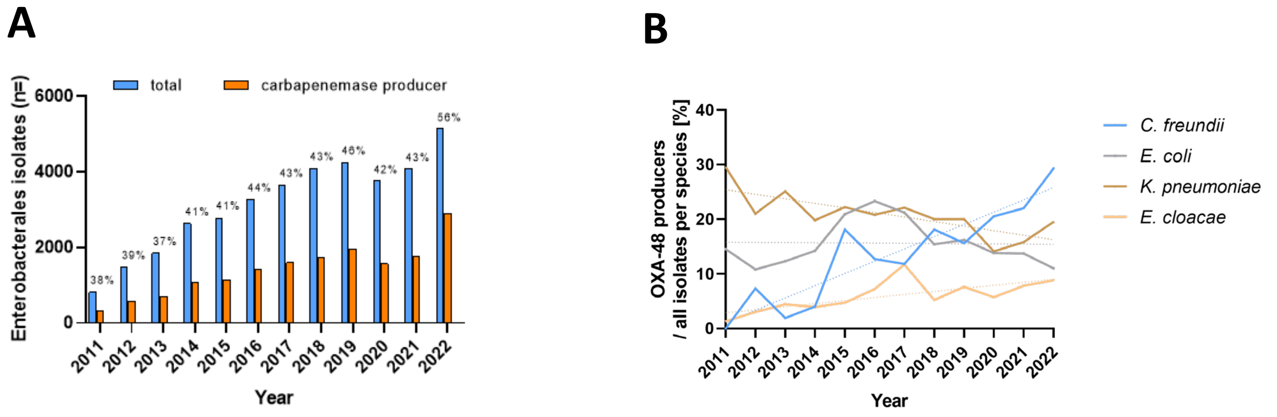

Supplementary Figure S1. Prevalence of carbapenemase-producing Enterobacterales in Germany 2011 to 2022. Number of total (blue) and carbapenemase-positive (orange) Enterobacterales isolates sent to the National Reference Center for Gram-negative bacteria (NRC) between 01/2011 and 12/2022 (A). Frequency of OXA-48 among the four most prevalent Enterobacterales species sent to the NRC between 01/2011 and 12/2022 (B).

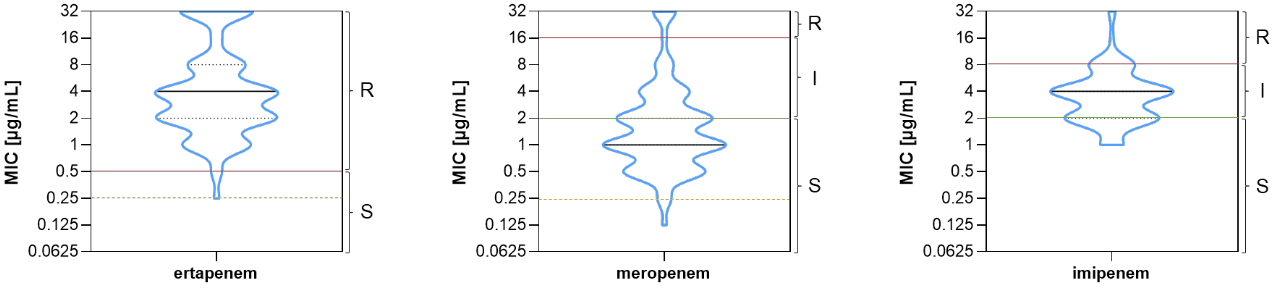

Supplementary Figure S2. Distribution of minimal inhibitory concentration (MIC) for ertapenem, meropenem and imipenem in *Citrobacter* sp. isolates producing OXA-48 and OXA-48-like carbapenemases (n=91). Median and quartile of the MICs are indicated by black lines. Breakpoints for resistant (R, red horizontal lines), susceptible with increased exposure (I) and susceptible (S, green horizontal lines) are indicated. The EUCAST screening cut-off values for suspected carbapenemase production in Enterobacterales are indicated for ertapenem and meropenem (yellow horizontal lines). In all isolates with lower carbapenem MICs (either ertapenem or meropenem MIC <0.5 mg/L respectively or meropenem MIC <1 mg/L and ertapenem MIC <2 mg/L), OXA-48 could be detected using the modified CIM test and lateral flow testing (Carba-5, NG Biotech).

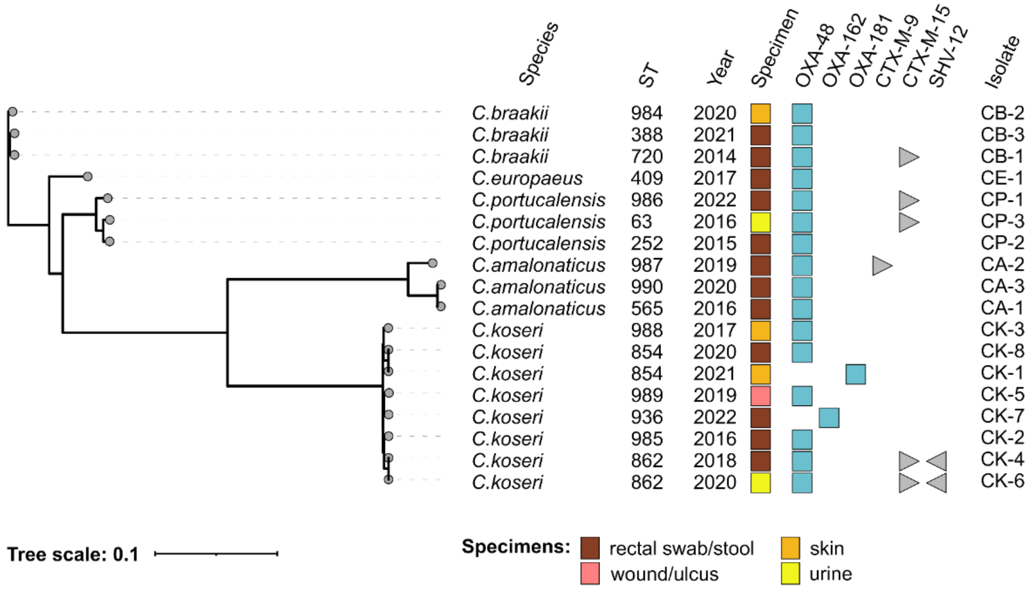

Supplementary Figure S3. SNP-based phylogeny of OXA-48 and OXA-48-like carbapenemase producing *C. braakii*, *C. europaeus*, *C. portucalensis* and *C. koseri* (n=18). Species, ST, year of isolation, specimen type, beta-lactamase resistance genes and isolate are shown. The scale bar indicates the number of substitutions per site.

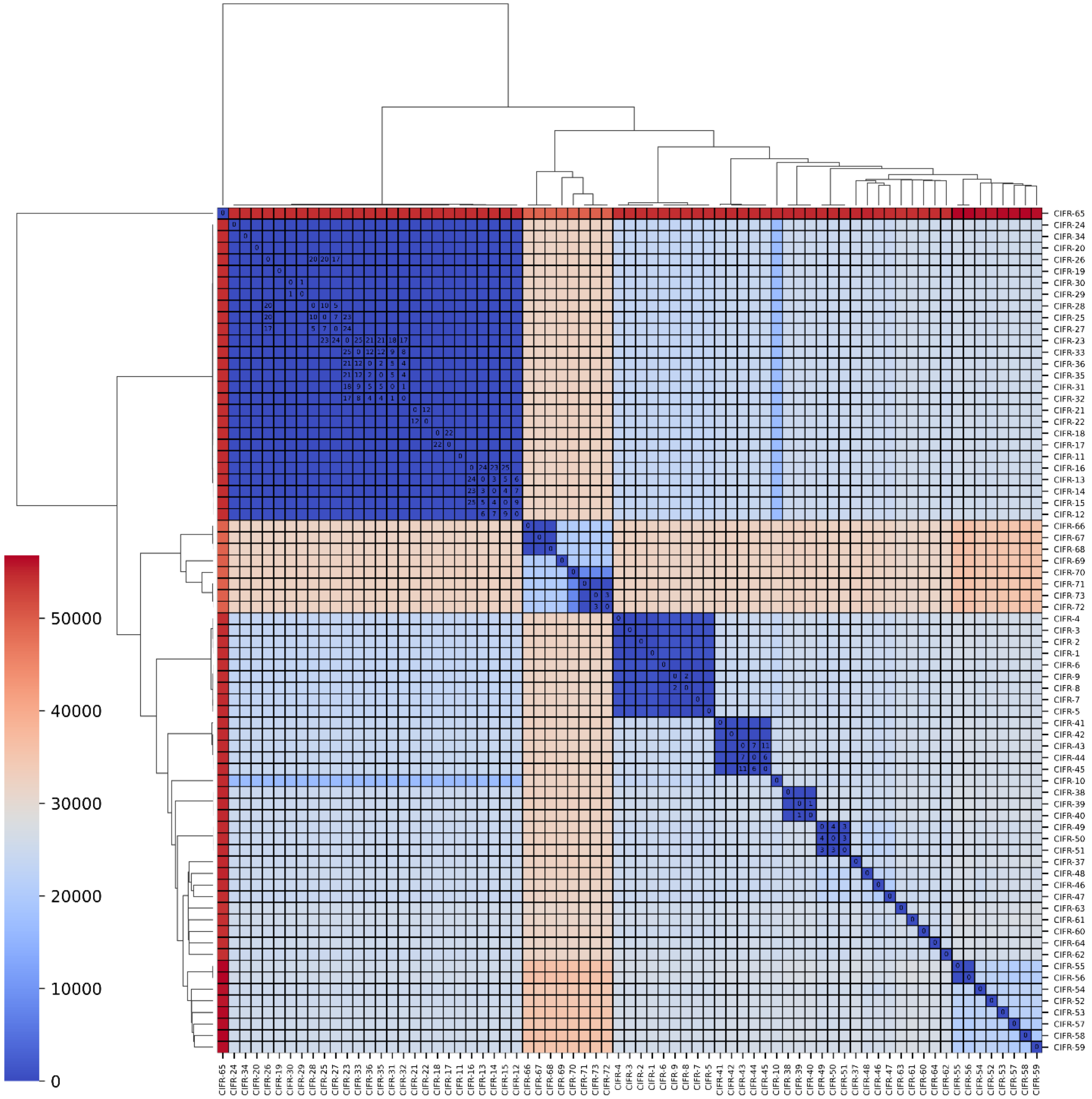

Supplementary Figure S4. SNP matrix of the core genome of 73 *C. freundii* isolates. For each isolate, the number of SNP differences with all other isolates was calculated and used to cluster isolates according to the Euclidean distance. The colour represents the SNP difference between isolates. Values of ≤25 SNP, indicating high similarity, are shown.

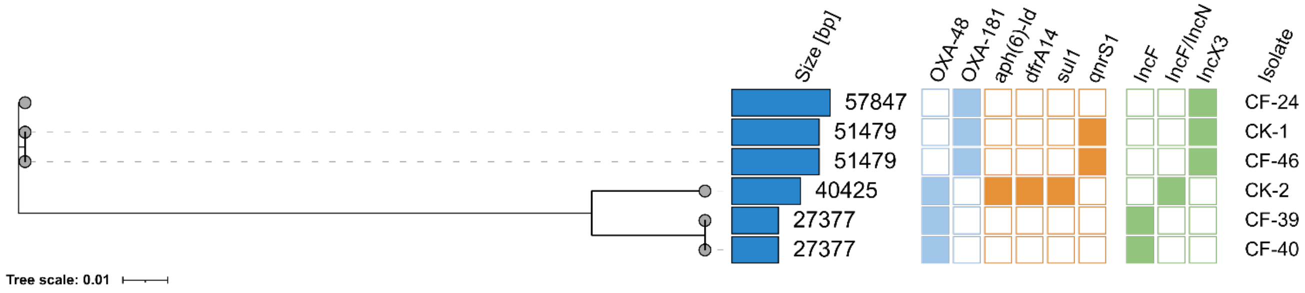

Supplementary Figure S5: Phylogeny of IncF and IncX3 OXA-48-like producing plasmids from *Citrobacter* spp. Phylogenetic tree of six plasmids. For each plasmid, its length, antibiotic resistance genes, Inc type and harbouring isolate are shown. The three IncX3 plasmids bearing *bla*$_{OXA181}$ share the previously described ~31 kb plasmid backbone of pEC21-OXA-181 (MG893567)[13]. Two identical IncF plasmids (27,377 bp), harbouring *bla*$_{OXA-48}$ have 95% sequence identity to previously described IncFII plasmid pLAU-OXA-48 [14]. The third IncF plasmid, from the isolate CK-2 has 72% sequence coverage and 99% nucleotide identity to pRHB38-C24_3, an IncN plasmid from *E. coli* [15]. The scale bar indicates the number of substitutions per site. CF, *Citrobacter freundii*; CK, *Citrobacter koseri*.

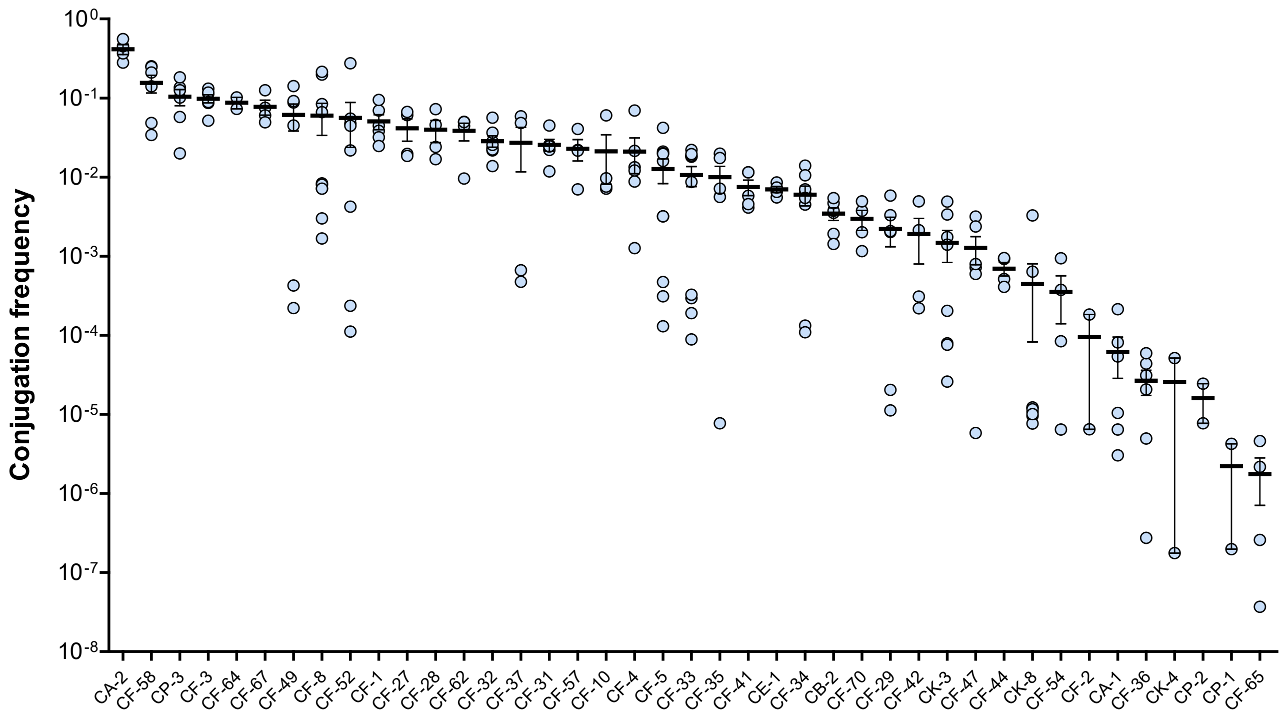

Supplementary Figure S6. Conjugation frequencies during HGT of plasmids harbouring *bla*$_{OXA-48}$ from *Citrobacter* spp. clinical isolates to *E. coli* J53. Horizontal bars represent the mean of at least three independent experiments. Error bars represent the standard error of the mean. CF, *Citrobacter freundii*; CK, *Citrobacter koseri*; CB, *Citrobacter braakii*; CA, *Citrobacter amalonaticus*; CE, *Citrobacter europaeus*; CP, *Citrobacter portucalensi*

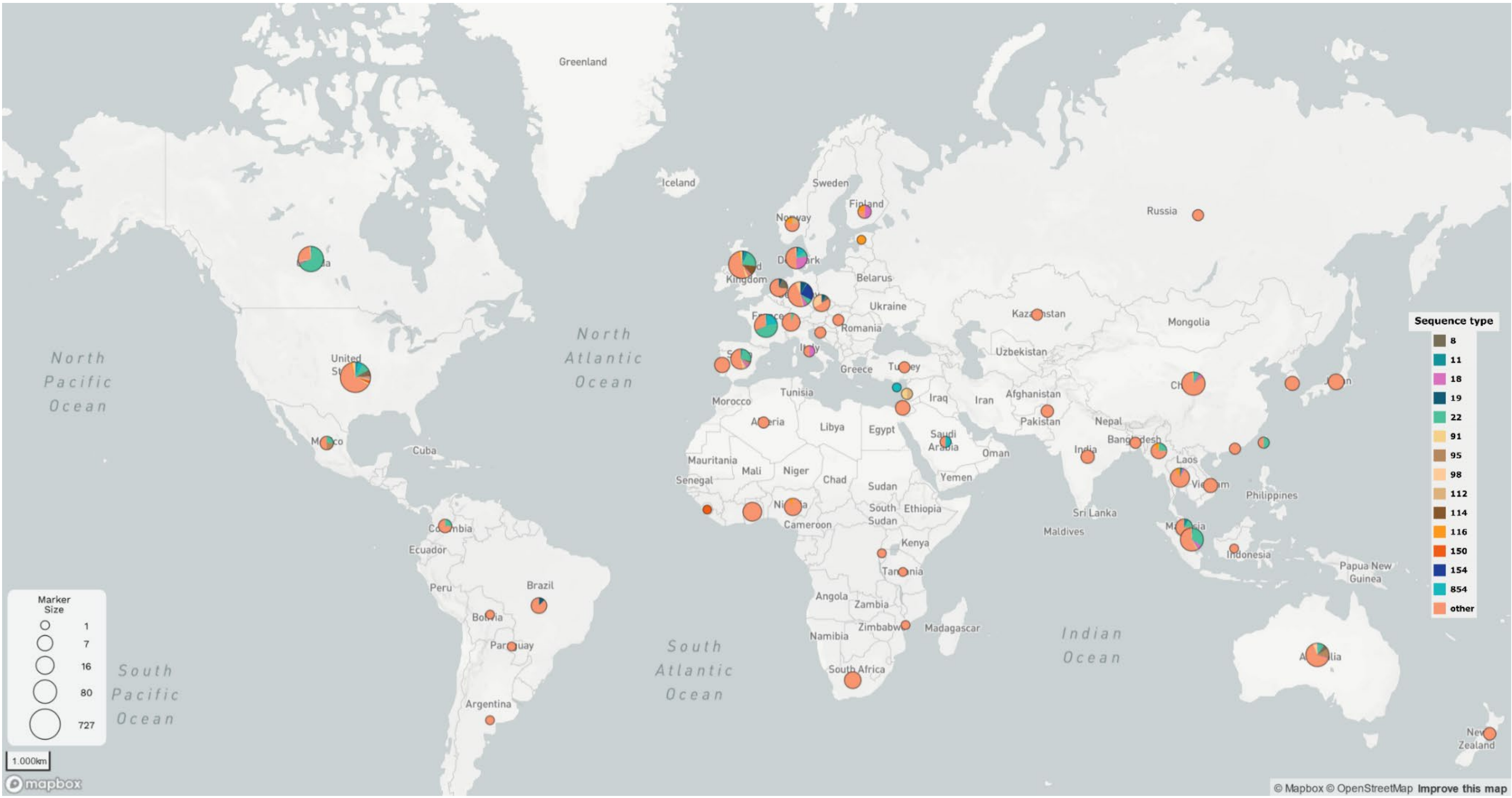

Supplementary Figure S7. Global epidemiology of *Citrobacter* spp. isolates. Geographical distribution of *Citrobacter* spp. isolates globally (n=1,946). Pie chart size is proportional to the numbers of isolates in each country. The colour code indicates the sequence types. Only sequence types with 25 or more isolates are shown, other sequence types are indicated as "other". The dataset is available online at the following link: https://microreact.org/project/6FCoZnFEQKmFGZowNwZGrw-global-epidemiology-of-citrobacter-sp

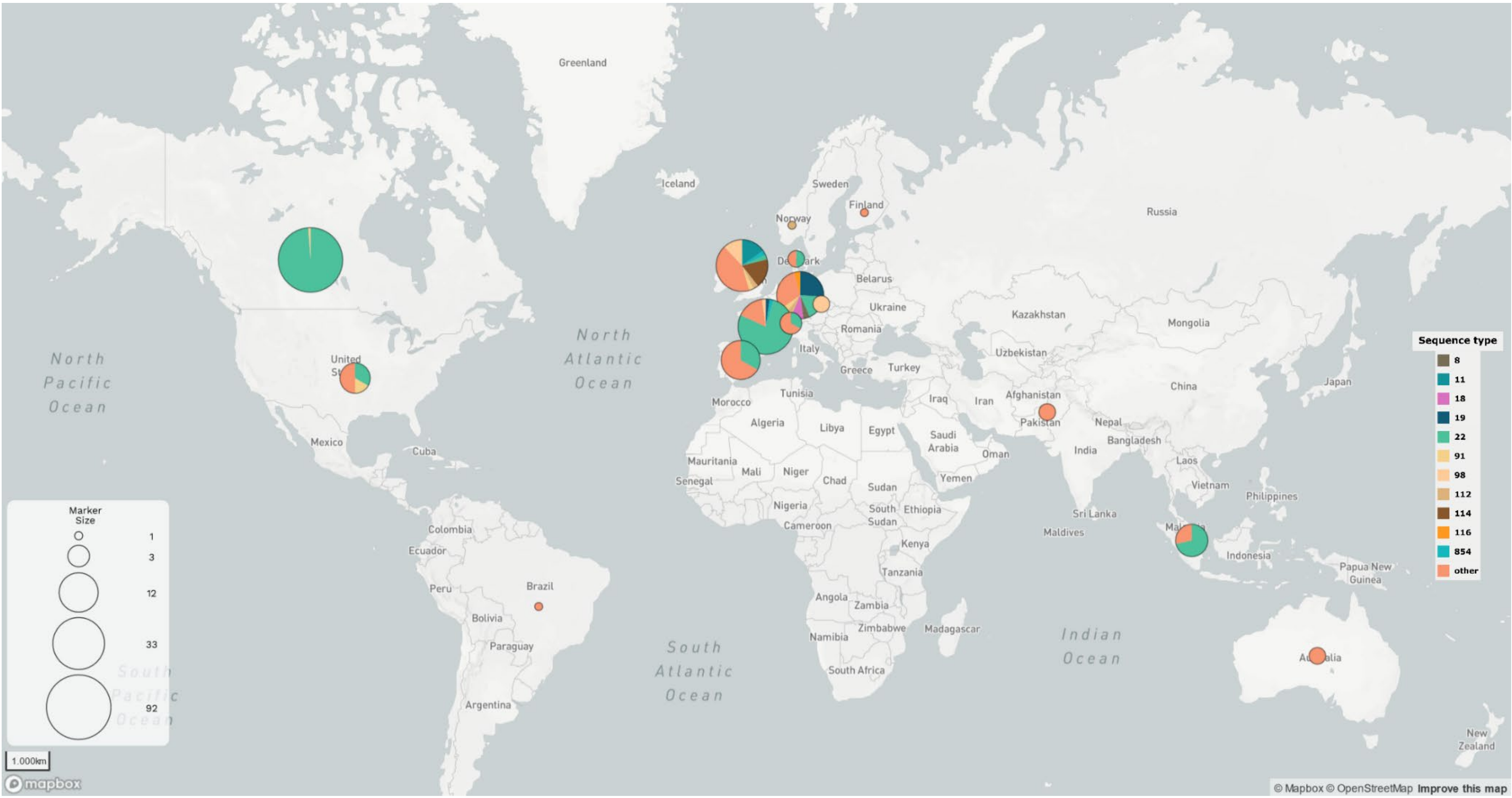

Supplementary Figure S8. Global epidemiology of *Citrobacter* spp. isolates producing OXA-48-like carbapenemases (n=220). Pie chart size is proportional to the numbers of isolates in each country. The colour code indicates the sequence types. Only sequence types with 25 or more isolates in the complete isolate collection are shown, other sequence types are indicated as "other". The dataset is available online at the following link: https://microreact.org/project/6FCoZnFEQKmFGZowNwZGrw-global-epidemiology-of-citrobacter-sp

Supplementary Table S1. Genetic characteristics of *Citrobacter* spp. isolates producing OXA-48-like carbapenemases. Antibiotic resistance genes were identified in assemblies using the Software ABRicate (https://github.com/tseemann/abricate) and the NCBI AMRFinderPlus database (Supplementary Methods). A cut-off of 100% gene coverage and 98% sequence identity was applied. Antibiotic resistance genes with less than 100% sequence identity compared to the identified gene in the database, are marked with an asterisk (*).

Isolate	Species	Acc. No.	Total length [Mbp]	N50 [Mbp]	GC (%)	Contigs n=	Beta-lactamase genes	Other antibiotic resistance genes	OXA-48-like plasmid type	Plasmid length [bp]
CA-1	*Citrobacter amalonaticus*	JAVTOL000000000	5.00	4,81	53.33	3	*bla*SED*, *bla*OXA-48	oqxB4*, oqxA10*	IncL	63,589
CA-2	*Citrobacter amalonaticus*	JAVTOL000000000	5.50	5,08	53.22	4	*bla*SED*, *bla*CTX-M-9, *bla*OXA-48	ant(2'')-Ia, aadA2, sul1, qnrA1*, sul1, sul1, aadA2, dfrA16, catA1, mcr-9.1	IncL	63,589
CA-3	*Citrobacter amalonaticus*	JAVTOM000000000	5.23	4,53	52.94	16	*bla*SED*, *bla*DHA-1, *bla*TEM-1, *bla*OXA-48	oqxA10*, oqxB20*, aac(6')-IIc, aac(3)-II, arr-269927220, ere(A)*, sul1, qnrB4, sul1, aac(6')-Ib*, catA2, tet(D)*, mcr-9.1, aph(6)-Id, aph(3'')-Ib, dfrA19	IncL	63,589
CB-1	*Citrobacter braakii*	JAVTON000000000	5.48	4,85	51.91	19	*bla*CMY-101*, *bla*OXA-1, *bla*OXA-48, *bla*CTX-M-15	aac(6')-Ib-cr*, dfrA1*, aadA1*, sul1, tet(A)*, mph(A)*, dfrA17*, aadA5, sul1, tet(A)*	IncL	63,589
CB-2	*Citrobacter braakii*	JAVTOO000000000	5.40	5,08	51.74	4	*bla*CMY-101*, *bla*OXA-48	qnrB72*, ant(2'')-Ia, aadA2, sul1, qnrA1*, sul1, ant(2'')-Ia, aadA2, sul1	IncL	77,709
CB-3	*Citrobacter braakii*	JAVTOP000000000	5.75	5,07	51.70	23	*bla*CMY-82*, *bla*TEM-1, *bla*TEM-1, *bla*CTX-M-14, *bla*TEM-1, *bla*OXA-48, *bla*OXA-427*	aph(6)-Id, aph(3'')-Ib*, qnrS1, mph(A)*, sul1, aadA2, dfrA12, aph(6)-Id, aac(6')-Ib4, sul1	IncL	63,589
CE-1	*Citrobacter europaeus*	JAVTOQ000000000	5.38	5,31	52.16	3	*bla*CFE-1*, *bla*OXA-2, *bla*OXA-48	qnrB27*, aph(3'')-Ib, aph(6)-Id, ant(2'')-Ia, sul1	IncL	63,589
CF-1	*Citrobacter freundii*	JAVTOR000000000	5.13	3,30	51.72	16	*bla*TEM-1, *bla*CTX-M-15, *bla*OXA-1, *bla*CMY-48, *bla*OXA-48	sul1, aadA1*, dfrA1*, sul2, aph(3'')-Ib*, aph(6)-Id, aac(3)-IIa*, aac(6')-Ib-cr*, qnrB1, tet(A)*, tet(A), dfrA14	IncL	62,812
CF-2	*Citrobacter freundii*	JAVTPD000000000	5.67	1,70	51.63	24	*bla*CMY-48, *bla*CTX-M-1, *bla*OXA-1, *bla*TEM-219*, *bla*TEM-150*, *bla*OXA-48, *bla*DHA-1, *bla*TEM-150*	tet(A), mph(A)*, dfrA1*, aadA1*, sul1, mph(A)*, sul1, arr-3, catB3, aac(6')-Ib-cr*, aac(3)-IId, sul1, qnrB4, sul1	IncL	70,452
CF-3	*Citrobacter freundii*	JAVTPO000000000	5.67	5,30	51.30	4	*bla*CMY-48, *bla*TEM-1, *bla*SHV-12, *bla*OXA-48	tet(A), sul1, aadA1*, dfrA1*, catA2, mcr-9.1, qnrA1*, sul1, sul2	IncL	62,827

Isolate	Species	Acc. No.	Total length [Mbp]	N50 [Mbp]	GC (%)	Contigs n=	Beta-lactamase genes	Other antibiotic resistance genes	OXA-48-like plasmid type	Plasmid length [bp]
CF-4	*Citrobacter freundii*	JAVTPZ000000000	5.28	5,15	51.65	6	*bla*CMY-48, *bla*OXA-48, *bla*SHV-12, *bla*BEL-1*, *bla*BEL-1*	tet(A), sul1, aadA1*, dfrA1*, qnrS1	IncL	63,589
CF-5	*Citrobacter freundii*	JAVTQK000000000	5.62	5,19	51.76	7	*bla*CMY-48, *bla*OXA-1, *bla*CTX-M-15, *bla*TEM-1, *bla*OXA-48	dfrA1, sat2, aadA1, tet(D)*, aac(6')-Ib-cr*	IncL	64,366
CF-6	*Citrobacter freundii*	JAVTQV000000000	5.97	5,26	51.85	6	*bla*CMY-48, *bla*CTX-M-15, *bla*OXA-1, *bla*TEM-1, *bla*TEM-1, *bla*OXA-1, *bla*OXA-48	aadA1, sat2, dfrA1, catA2, sul2, aph(3'')-Ib*, aph(6)-Id, catA2, dfrA14, aac(6')-Ib-cr*, catB3, arr-3, sul1, mph(A)*, aac(3)-IId, aac(6')-Ib-cr*	IncL	72,399
CF-7	*Citrobacter freundii*	JAVTRG000000000	5.71	5,15	51.64	5	*bla*CTX-M-3, *bla*CTX-M-3, *bla*CMY-48, *bla*OXA-1, *bla*TEM-1, *bla*OXA-48	aadA1, sat2, dfrA1, aac(6')-Ib-cr*, catB3, arr-3, sul1, mph(A)*, aac(3)-IId, tet(D)*, sul2	IncL	74,309
CF-8	*Citrobacter freundii*	JAVTRL000000000	5.43	5,18	51.82	6	*bla*CMY-48, *bla*OXA-1, *bla*TEM-1, *bla*OXA-48	aadA1, sat2, dfrA1, aac(6')-Ib-cr*, catB3, arr-3, sul1, mph(A)*, aac(3)-IId, qnrB19, tet(D)*, sul2, catA2	IncL	71,166
CF-9	*Citrobacter freundii*	JAVTRM000000000	5.39	5,18	51.78	6	*bla*CMY-48, *bla*OXA-1, *bla*OXA-48	aadA1, sat2, dfrA1, aac(6')-Ib-cr*, catB3, arr-3, sul1, mph(A)*	IncL	63,126
CF-10	*Citrobacter freundii*	JAVTOT000000000	5.44	5,12	51.58	18	*bla*CMY-152, *bla*OXA-48		IncL	71,082
CF-11	*Citrobacter freundii*	JAVTOU000000000	5.23	5,02	51.85	6	*bla*CMY-152, *bla*OXA-162		IncL	63,412
CF-12	*Citrobacter freundii*	JAVTOV000000000	5.15	4,98	51.73	5	*bla*CTX-M-3, *bla*CTX-M-3, *bla*CMY-152, *bla*OXA-48	aph(6)-Id, aph(3'')-Ib, aadA1, sat2, dfrA1	IncL	48,617
CF-13	*Citrobacter freundii*	JAVTOW000000000	5.61	5,05	51.35	22	*bla*CTX-M-3, *bla*CMY-152, *bla*OXA-48, *bla*CTX-M-9	aadA2, dfrA16, sul1, aph(6)-Id, aph(3'')-Ib, aadA1, sat2, dfrA1, ant(2'')-Ia, aadA2, sul1, qnrA1*, sul1, tet(A), mcr-9.1	IncL	62,812
CF-14	*Citrobacter freundii*	JAVTOX000000000	5.61	5,05	51.36	6	*bla*CTX-M-3, *bla*CTX-M-3, *bla*CMY-152, *bla*CTX-M-9, *bla*OXA-48	aph(6)-Id, aph(3'')-Ib, aadA1, sat2, dfrA1, ant(2'')-Ia, aadA2, sul1, qnrA1*, sul1, tet(A), dfrA16, aadA2, sul1, mcr-9.1	IncL	62,812
CF-15	*Citrobacter freundii*	JAVTOY000000000	5.49	5,30	51.45	5	*bla*CTX-M-9, *bla*CTX-M-3, *bla*CTX-M-3, *bla*CMY-152, *bla*OXA-48	sul1, aadA2, ant(2'')-Ia, mcr-9.1, aph(6)-Id, aph(3'')-Ib, aadA1, sat2, dfrA1	IncL	62,812

Isolate	Species	Acc. No.	Total length [Mbp]	N50 [Mbp]	GC (%)	Contigs n=	Beta-lactamase genes	Other antibiotic resistance genes	OXA-48-like plasmid type	Plasmid length [bp]
CF-16	*Citrobacter freundii*	JAVTOZ000000000	5.42	4,99	51.38	16	*bla*CMY-152, *bla*CTX-M-3, *bla*CTX-M-9, *bla*OXA-48	dfrA1, sat2, aadA1, aph(3'')-Ib, aph(6)-Id, mcr-9.1, ant(2'')-Ia, aadA2, sul1, mcr-9.1	IncL	62,722
CF-17	*Citrobacter freundii*	JAVTPA000000000	5.11	4,83	51.82	18	*bla*CMY-152, *bla*OXA-48	sul1, aph(3')-Ia, aadA2, sul1, qnrA1*, sul1, aadA2, ant(2'')-Ia, mph(A)*, dfrA12, aph(3'')-Ib, aph(6)-Id	IncL	63,589
CF-18	*Citrobacter freundii*	JAVTPB000000000	5.07	4,89	51.74	4	*bla*OXA-48, *bla*CMY-152		IncL	63,589
CF-19	*Citrobacter freundii*	JAVTPC000000000	5.45	4,92	51.63	16	*bla*TEM-1, *bla*CMY-152, *bla*SHV-12, *bla*TEM-1, *bla*OXA-48	dfrA19, aadA2, sul1, aph(3')-Ia, catA1*, aph(6)-Id, aph(3'')-Ib, sul2*, mcr-9.1, aac(3)-II, arr-269927220, ere(A)*, sul1, sul2, catA2, qnrB2, aph(6)-Id, aph(3'')-Ib	IncL	64,366
CF-20	*Citrobacter freundii*	JAVTPE000000000	5.58	5,10	51.53	12	*bla*CMY-152*, *bla*TEM-1, *bla*OXA-10, *bla*SHV-12, *bla*TEM-1, *bla*OXA-48	aph(6)-Id*, aph(3'')-Ib*, sul2*, catA1*, mph(A)*, sul1, aadA2, dfrA12, tet(D)*, ant(2'')-Ia, catB8, aadA1*, sul1, catA1*, tet(B), sul2, sul1, ere(A)*, arr-269927220, aac(3)-II, aac(6')-IIc, aph(6)-Id, aph(3'')-Ib*, sul2	IncL	63,589
CF-21	*Citrobacter freundii*	JAVTPF000000000	5.84	3,76	51.45	30	*bla*TEM-1, *bla*CTX-M-15*, *bla*OXA-1, *bla*CMY-152*, *bla*OXA-48	catA1*, sul2*, aph(3'')-Ib*, aph(6)-Id, aac(3)-IIa*, aac(6')-Ib-cr*	IncL	63,589
CF-22	*Citrobacter freundii*	JAVTPG000000000	5.51	5,09	51.70	4	*bla*TEM-1, *bla*CTX-M-15*, *bla*OXA-1, *bla*CMY-152*, *bla*OXA-48	catA1*, sul2*, aph(3'')-Ib*, aph(6)-Id, aac(3)-IIa*, aac(6')-Ib-cr*	IncL	63,589
CF-23	*Citrobacter freundii*	JAVTPH000000000	5.09	4,94	51.96	3	*bla*CMY-152*, *bla*OXA-10, *bla*VIM-1, *bla*TEM-1, *bla*OXA-162	tet(A), aadA1*, ant(2'')-Ia, sul1, dfrA10, sul1, aadA1*, aac(6')-Ib*	IncL	63,412
CF-24	*Citrobacter freundii*	JAVTPI000000000	5.18	4,71	51.74	5	*bla*TEM-1, *bla*CMY-152*, *bla*CTX-M-3, *bla*OXA-181	catA1*, sul2*, aph(3'')-Ib*, aph(6)-Id	IncX3	57,847
CF-25	*Citrobacter freundii*	JAVTPJ000000000	5.24	2,64	51.84	7	*bla*TEM-1, *bla*CMY-152*, *bla*CTX-M-3, *bla*OXA-48	catA1*, sul2*, aph(3'')-Ib*, aph(6)-Id, ant(2'')-Ia, aadA2, sul1, qnrA1*, sul1, aph(3')-Ia	IncL	71,166

Isolate	Species	Acc. No.	Total length [Mbp]	N50 [Mbp]	GC (%)	Contigs n=	Beta-lactamase genes	Other antibiotic resistance genes	OXA-48-like plasmid type	Plasmid length [bp]
CF-26	*Citrobacter freundii*	JAVTPK000000000	5.25	4,89	51.83	5	*bla*CTX-M-3, *bla*CTX-M-3, *bla*TEM-1, *bla*CMY-152*, *bla*OXA-48	catA1*, sul2*, aph(3'')-Ib*, aph(6)-Id, ant(2'')-Ia, aadA2, sul1, qnrA1*, sul1, aph(3')-Ia	IncL	71,166
CF-27	*Citrobacter freundii*	JAVTPL000000000	5.24	4,95	51.83	5	*bla*CTX-M-3, *bla*CTX-M-3, *bla*TEM-1, *bla*CMY-152*, *bla*OXA-48	catA1*, sul2*, aph(3'')-Ib*, aph(6)-Id, ant(2'')-Ia, aadA2, sul1, qnrA1*, sul1	IncL	71,166
CF-28	*Citrobacter freundii*	JAVTPM000000000	5.24	4,98	51.84	4	*bla*TEM-1, *bla*CMY-152*, *bla*CTX-M-3, *bla*OXA-48	catA1*, sul2*, aph(3'')-Ib*, aph(6)-Id, ant(2'')-Ia, aadA2, sul1, qnrA1*, sul1, aph(3')-Ia	IncL	71,160
CF-29	*Citrobacter freundii*	JAVTPN000000000	5.69	3,81	51.62	14	*bla*CMY-152*, *bla*TEM-1, *bla*TEM-1, *bla*OXA-48	aph(6)-Id, aph(3'')-Ib*, sul2*, catA1*, tet(A), mcr-9.1, aph(6)-Id, aph(3'')-Ib, dfrA19, sul1, ere(A)*, arr-269927220, aac(3)-II, aac(6')-IIc, aac(6')-Ib*, sul2	IncL	63,589
CF-30	*Citrobacter freundii*	JAVTPP000000000	5.72	5,16	51.64	10	*bla*TEM-1, *bla*CMY-152*, *bla*TEM-1, *bla*OXA-48	sul2, catA1*, tet(A), aph(3'')-Ib, aph(6)-Id, mcr-9.1, aph(6)-Id, aph(3'')-Ib, dfrA19, sul1, ere(A)*, arr-269927220, aac(3)-II, aac(6')-IIc, aac(6')-Ib*, sul2	IncL	63,589
CF-31	*Citrobacter freundii*	JAVTPQ000000000	5.33	2,17	51.50	14	*bla*CMY-152*, *bla*OXA-48, *bla*SHV-12, *bla*TEM-1	aac(6')-IIc, aac(3)-II, arr-269927220, ere(A)*, catA1*, sul2*, mcr-9.1, qnrA1*, aph(3'')-Ib, aph(6)-Id, dfrA19, catA2, sul1	IncL	63,589
CF-32	*Citrobacter freundii*	JAVTPR000000000	5.32	4,97	51.53	4	*bla*CMY-152*, *bla*OXA-48, *bla*SHV-12, *bla*TEM-1	ere(A)*, arr-269927220, aac(3)-II, aac(6')-IIc, catA1*, tet(D)*, sul2*, mcr-9.1, qnrA1*, aph(3'')-Ib, aph(6)-Id, dfrA19, catA2, sul1	IncL	63,589
CF-33	*Citrobacter freundii*	JAVTPS000000000	5.30	4,57	51.54	10	*bla*CMY-152*, *bla*TEM-1, *bla*SHV-12, *bla*OXA-48, *bla*TEM-1	catA1*, tet(D)*, catA2, aac(6')-IIc, aac(3)-II, arr-269927220, ere(A)*, sul1, dfrA19, sul2*, aph(3'')-Ib*, aph(6)-Id	IncL	63,588
CF-34	*Citrobacter freundii*	JAVTPT000000000	5.31	3,02	51.64	14	*bla*TEM-1, *bla*CMY-152*, *bla*TEM-1, *bla*SHV-12, *bla*OXA-48	catA1*, sul2*, aph(3'')-Ib*, aph(6)-Id, mcr-9.1, aph(6)-Id, aph(3'')-Ib, dfrA19, sul1, ere(A)*, arr-269927220, aac(3)-II, aac(6')-IIc	IncL	63,589

Isolate	Species	Acc. No.	Total length [Mbp]	N50 [Mbp]	GC (%)	Contigs n=	Beta-lactamase genes	Other antibiotic resistance genes	OXA-48-like plasmid type	Plasmid length [bp]
CF-35	*Citrobacter freundii*	JAVTPU000000000	5.33	4,86	51.63	5	*bla*CMY-152*, *bla*OXA-48, *bla*SHV-12, *bla*TEM-1	aac(6')-IIc, aac(3)-II, arr-269927220, ere(A)*, sul1, dfrA19, catA1*, sul2*, mcr-9.1, aph(3'')-Ib, aph(6)-Id, catA2	IncL	63,589
CF-36	*Citrobacter freundii*	JAVTPV000000000	5.08	4,90	51.89	7	*bla*TEM-1, *bla*CMY-152*, *bla*OXA-48	catA1*, sul2*, aph(3'')-Ib*, aph(6)-Id	IncL	63,589
CF-37	*Citrobacter freundii*	JAVTPW000000000	5.39	4,96	51.61	13	*bla*CMY-76*, *bla*SHV-12, *bla*TEM-1, *bla*OXA-48	fosA7*, sul2, catA2, tet(D)*, mcr-9.1, aph(6)-Id, aph(3'')-Ib, dfrA19, sul1, ere(A)*, arr-269927220, aac(3)-II, aac(6')-IIc, dfrA12, aadA2, sul1, mph(A)*, aac(6')-Ib*, sul1, aph(3')-Ia, aadA2	IncL	63,589
CF-38	*Citrobacter freundii*	JAVTPX000000000	5.52	4,99	51.60	6	*bla*TEM-1, *bla*CMY-117*, *bla*TEM-1, *bla*OXA-48	sul1, aadA5, dfrA17*, aac(3)-IIa	IncL	63,589
CF-39	*Citrobacter freundii*	JAVTPY000000000	5.27	5,08	51.85	4	*bla*TEM-1, *bla*CMY-117*, *bla*DHA-1, *bla*OXA-1, *bla*OXA-48	aadA5, dfrA17*, mph(A)*, sul1, qnrB4, sul1, arr-3, catB3, aac(6')-Ib-cr5	IncF	27,377
CF-40	*Citrobacter freundii*	JAVTQA000000000	5.56	5,04	51.79	5	*bla*TEM-1, *bla*CMY-117*, *bla*CTX-M-1, *bla*OXA-1, *bla*DHA-1, *bla*OXA-48	aadA5, dfrA17*, mph(A)*, aac(6')-Ib-cr5, catB3, arr-3, sul1, qnrB4, sul1, mph(A)*	IncF	27,377
CF-41	*Citrobacter freundii*	JAVTQB000000000	5.41	2,95	51.61	7	*bla*CMY-135*, *bla*OXA-48	dfrA1*, aadA1*, sul1, tet(A)	IncL	78,492
CF-42	*Citrobacter freundii*	JAVTQC000000000	5.33	4,97	51.54	10	*bla*CMY-2, *bla*CMY-135*, *bla*CMY-2, *bla*OXA-48, *bla*OXA-1, *bla*CTX-M-15, *bla*TEM-1	floR, tet(A), aph(6)-Id, aph(3'')-Ib*, sul2, tet(A), aac(6')-Ib-cr*, aac(3)-IIa*	IncL	71,166
CF-43	*Citrobacter freundii*	JAVTQD000000000	5.37	5,01	51.58	13	*bla*CMY-135*, *bla*OXA-10, *bla*CMY-2, *bla*OXA-162, *bla*OXA-1, *bla*CTX-M-15, *bla*TEM-1, *bla*NPS-1*	arr-2, cmlA5, aadA1*, sul1, armA, msr(E)*, mph(E), floR, tet(A), aph(6)-Id, aph(3'')-Ib*, sul2, tet(A), aac(6')-Ib-cr*, aac(3)-IIa*	IncL	63,412
CF-44	*Citrobacter freundii*	JAVTQE000000000	5.39	4,99	51.60	10	*bla*CMY-135*, *bla*OXA-10, *bla*OXA-1, *bla*CTX-M-15, *bla*TEM-1, *bla*CMY-2, *bla*OXA-162	arr-2, cmlA5, aadA1*, sul1, armA, msr(E)*, mph(E), floR, tet(A), aac(6')-Ib-cr*, aac(3)-IIa*, tet(A), aph(6)-Id, aph(3'')-Ib*, sul2, tet(A)	IncL	67,081

Isolate	Species	Acc. No.	Total length [Mbp]	N50 [Mbp]	GC (%)	Contigs n=	Beta-lactamase genes	Other antibiotic resistance genes	OXA-48-like plasmid type	Plasmid length [bp]
CF-45	*Citrobacter freundii*	JAVTQF000000000	5.36	5,03	51.56	5	*bla*CMY-135*, *bla*OXA-10, *bla*CMY-2, *bla*OXA-162, *bla*OXA-1, *bla*CTX-M-15, *bla*TEM-1	arr-2, cmlA5, aadA1*, sul1, armA, msr(E)*, mph(E), floR, tet(A), aph(6)-Id, aph(3'')-Ib*, sul2, tet(A), aac(6')-Ib-cr*, aac(3)-IIa*	IncL	63,412
CF-46	*Citrobacter freundii*	JAVTQG000000000	5.85	3,00	51.47	34	*bla*CMY-48, *bla*CTX-M-9, *bla*CTX-M-15, *bla*OXA-1, *bla*TEM-1, *bla*OXA-181	ant(2'')-Ia, aadA2, sul1, dfrA16, aadA2, sul1, qnrA1*, sul1, mcr-9.1, aac(3)-IIa*, aac(6')-Ib-cr*, aadA6*, ant(2'')-Ia, qnrS1	IncX3	51,479
CF-47	*Citrobacter freundii*	JAVTQH000000000	5.34	4,99	51.76	4	*bla*CTX-M-15, *bla*CMY-76*, *bla*OXA-1, *bla*CTX-M-15, *bla*TEM-1, *bla*OXA-48	aac(6')-If, fosA7*, dfrA14, qnrB1, aac(6')-Ib-cr*, aac(3)-IIa*, aph(6)-Id, aph(3'')-Ib*, sul2	IncL	56,518
CF-48	*Citrobacter freundii*	JAVTQI000000000	4.97	3,05	51.82	18	*bla*CMY-48*, *bla*OXA-48		IncL	63,589
CF-49	*Citrobacter freundii*	JAVTQJ000000000	5.55	5,20	51.54	5	*bla*CMY-78, *bla*CTX-M-9, *bla*OXA-162	fosA7*, ant(2'')-Ia, aadA2, sul1, qnrA1*, sul1, mcr-9.1	IncL	63,412
CF-50	*Citrobacter freundii*	JAVTQL000000000	5.65	4,05	51.43	16	*bla*CMY-78, *bla*CTX-M-9, *bla*OXA-162	fosA7*, ant(2'')-Ia, aadA2, sul1, qnrA1*, sul1, mcr-9.1	IncL	63,412
CF-51	*Citrobacter freundii*	JAVTQM000000000	5.63	5,19	51.43	5	*bla*CMY-78, *bla*CTX-M-9, *bla*OXA-162	fosA7*, ant(2'')-Ia, aadA2, sul1, qnrA1*, sul1, mcr-9.1	IncL	63,412
CF-52	*Citrobacter freundii*	JAVTQN000000000	5.33	4,64	51.77	4	*bla*CMY-79*, *bla*OXA-48		IncL	64,783
CF-53	*Citrobacter freundii*	JAVTQO000000000	5.03	4,97	51.71	2	*bla*CMY-79, *bla*OXA-48		IncL	63,675
CF-54	*Citrobacter freundii*	JAVTQP000000000	5.42	4,80	51.46	12	*bla*CMY-75*, *bla*TEM-1, *bla*SHV-12, *bla*OXA-48	catA2, aadA2, sul1, qnrA1*, sul1, dfrA19, aph(3'')-Ib, aph(6)-Id, mcr-9.1, tet(D)*, aac(6')-IIc, aac(3)-II, arr-269927220, ere(A)*, sul1, sul2	IncL	63,589
CF-55	*Citrobacter freundii*	JAVTQQ000000000	5.29	4,97	51.67	5	*bla*CMY-110*, *bla*OXA-48	tet(A)*, sul1, aadA1*, dfrA1*	IncL	63,589
CF-56	*Citrobacter freundii*	JAVTQR000000000	5.27	4,96	51.69	5	*bla*OXA-48, *bla*CMY-110*	tet(A)*, sul1, aadA1*, dfrA1*	IncL	63,590
CF-57	*Citrobacter freundii*	JAVTQS000000000	5.19	5,09	51.56	3	*bla*CMY-51*, *bla*OXA-48	aac(6')-Ib-cr*, arr-3, dfrA27, aadA16, sul1, tet(A)	IncL	62,274
CF-58	*Citrobacter freundii*	JAVTQT000000000	5.44	4,98	51.62	6	*bla*CMY-48*, *bla*TEM-1*, *bla*OXA-48	tet(B), aac(3)-IId, catA2, sul2, aph(3'')-Ib*, aph(6)-Id, tet(A), tet(A), mph(A)*, sul1, qnrB6*,	IncL	63,589

Isolate	Species	Acc. No.	Total length [Mbp]	N50 [Mbp]	GC (%)	Contigs n=	Beta-lactamase genes	Other antibiotic resistance genes	OXA-48-like plasmid type	Plasmid length [bp]
								sul1, aadA16, dfrA27, arr-3, aac(6')-Ib-cr*		
CF-59	*Citrobacter freundii*	JAVTQU000000000	5.34	5,13	51.67	4	*bla*CMY-97*, *bla*TEM-1, *bla*OXA-48	mph(A)*, sul1, aadA5, dfrA17*, sul2*, aph(3'')-Ib*, aph(6)-Id, aph(3')-Ia	IncL	64,586
CF-60	*Citrobacter freundii*	JAVTQW000000000	5.62	5,09	51.66	5	*bla*CMY-89*, *bla*TEM-110, *bla*TEM-110, *bla*OXA-162	aac(6')-If*, sul1, ere(A)*, arr-269927220, aac(3)-II, aac(6')-IIc, mcr-9.1, aph(3')-Ia, aph(6)-Id, aph(3'')-Ib, dfrA19, sul1	IncL	63,412
CF-61	*Citrobacter freundii*	JAVTQX000000000	5.18	5,06	51.99	4	*bla*CMY-75, *bla*OXA-162, *bla*TEM-150*, *bla*OXA-1	qnrS2, aac(6')-Ib-cr5, catB3, arr-3, sul1, mph(A)*, ant(2'')-Ia, sul1	IncL	63,412
CF-62	*Citrobacter freundii*	JAVTQY000000000	5.37	4,88	51.46	11	*bla*CTX-M-9, *bla*OXA-48, *bla*CMY-81	sul1, aadA2, ant(2'')-Ia, mcr-9.1	IncL	62,812
CF-63	*Citrobacter freundii*	JAVTQZ000000000	5.01	4,95	51.87	2	*bla*CMY-75*, *bla*OXA-48		IncL	62,812
CF-64	*Citrobacter freundii*	JAVTRA000000000	5.34	4,88	51.41	4	*bla*CMY-135*, *bla*CTX-M-9, *bla*OXA-48	aac(6')-If, ant(2'')-Ia, aadA2, sul1, qnrA1*, sul1, tet(A), dfrA16, aadA2, sul1	IncL	71,166
CF-65	*Citrobacter freundii*	JAVTRB000000000	5.76	5,34	51.74	6	*bla*CMY-76*, *bla*OXA-48, *bla*CTX-M-3	qnrB38*, dfrA14	IncL	62,811
CF-66	*Citrobacter freundii*	JAVTRC000000000	5.28	4,90	51.54	3	*bla*CMY-109, *bla*OXA-1, *bla*OXA-48	qnrB38*, mcr-9.1, sul1, arr-3, catB3, aac(6')-Ib-cr5	IncL	46,786
CF-67	*Citrobacter freundii*	JAVTRD000000000	5.26	4,96	51.88	12	*bla*CMY-109, *bla*OXA-48, *bla*TEM-1	qnrB38*, sul1, aadA2, dfrA12, aph(3'')-Ib, aph(6)-Id, tet(A), sul2, aph(3')-Ia, mph(A)*, aac(3)-IId, mph(A)*	IncL	62,812
CF-68	*Citrobacter freundii*	JAVTRE000000000	5.80	3,33	51.61	19	*bla*CMY-109, *bla*VIM-2, *bla*CTX-M-15, *bla*CMY-4, *bla*TEM-1, *bla*OXA-1, *bla*CTX-M-15, *bla*OXA-48	qnrB38*, aph(3')-Ib, sul1, aac(6')-Ib4, floR, tet(A), aph(6)-Id, aph(3'')-Ib*, sul2, dfrA14, aac(6')-Ib-cr*, qnrB32*	IncL	66,482
CF-69	*Citrobacter freundii*	JAVTRF000000000	4.96	4,90	51.91	3	*bla*CMY-150*, *bla*OXA-48	qnrB38*, aac(6')-If*	IncL	59,247
CF-70	*Citrobacter freundii*	JAVTRH000000000	5.28	4,89	51.77	4	*bla*OXA-48, *bla*CMY-150*	aac(6')-If, qnrB38*	IncL	62,812
CF-71	*Citrobacter freundii*	JAVTRI000000000	5.35	4,99	51.84	5	*bla*CMY-150*, *bla*OXA-1, *bla*CTX-M-15, *bla*OXA-48	qnrB38, dfrA12, aadA2, sul1, mph(A)*, aac(6')-Ib-cr*	IncL	63,589

Isolate	Species	Acc. No.	Total length [Mbp]	N50 [Mbp]	GC (%)	Contigs n=	Beta-lactamase genes	Other antibiotic resistance genes	OXA-48-like plasmid type	Plasmid length [bp]
CF-72	*Citrobacter freundii*	JAVTRJ000000000	5.45	5,12	51.79	8	*bla*CMY-150*, *bla*CTX-M-15, *bla*OXA-48, *bla*TEM-1	qnrB38, aac(6')-Ib-cr*, arr-3, dfrA27, aadA16, sul1, mph(A)*	IncL	67,258
CF-73	*Citrobacter freundii*	JAVTRK000000000	5.45	2,82	51.79	17	*bla*CMY-150*, *bla*CTX-M-15, *bla*TEM-1, *bla*OXA-48	qnrB38, aac(6')-Ib-cr*, arr-3, dfrA27, aadA16, sul1, qnrB6*, sul1, mph(A)*	IncL	67,258
CK-1	*Citrobacter koseri*	JAVTRN000000000	5.05	2,87	53.64	7	*bla*MAL-1, *bla*OXA-181	fosA7*, qnrS1	IncX3	51,479
CK-2	*Citrobacter koseri*	JAVTRO000000000	5.11	4,71	53.30	3	*bla*MAL-1*, *bla*OXA-48	sul2, dfrA14, aph(6)-Id	IncF/IncN	40,425
CK-3	*Citrobacter koseri*	JAVTRP000000000	4.81	4,70	53.67	4	*bla*MAL-2*, *bla*OXA-48	fosA7*	IncL	63,489
CK-4	*Citrobacter koseri*	JAVTRQ000000000	5.09	4,52	53.40	14	*bla*CKO-1*, *bla*SHV-12, *bla*TEM-1, *bla*OXA-48, *bla*CTX-M-15, *bla*TEM-1	aph(3')-Ia, aph(6)-Id, aph(3'')-Ib*, mcr-9.1, sul2, sul1, aph(6)-Id, aph(3'')-Ib*, dfrA19, sul1, ere(A)*, arr-269927220, aac(3)-II, aac(6')-IIc, sul2	IncL	61,955
CK-5	*Citrobacter koseri*	JAVTRR000000000	5.19	4,86	53.24	5	*bla*MAL-1*, *bla*OXA-48		IncL	58,879
CK-6	*Citrobacter koseri*	JAVTRS000000000	5.08	4,66	53.40	26	*bla*CKO-1*, *bla*TEM-1, *bla*OXA-48, *bla*SHV-12, *bla*CTX-M-15, *bla*TEM-1	aph(3')-Ia, aph(6)-Id, aph(3'')-Ib*, sul1, dfrA19, sul1, ere(A)*, arr-269927220, aac(3)-II, aac(6')-IIc, mcr-9.1, sul2	IncL	61,966
CK-7	*Citrobacter koseri*	JAVTRT000000000	4.69	4,63	53.85	2	*bla*CKO-1*, *bla*OXA-162	fosA7*	IncL	63,412
CK-8	*Citrobacter koseri*	JAVTRU000000000	4.90	4,83	53.71	3	*bla*MAL-1, *bla*OXA-48	fosA7*	IncL	65,289
CP-1	*Citrobacter portucalensis*	JAVTRV000000000	5.57	2,96	51.66	22	*bla*CMY-2, *bla*OXA-1, *bla*TEM-1, *bla*TEM-1, *bla*OXA-48, *bla*CTX-M-15	qnrB32, aac(6')-Ib-cr*, aac(3)-IIa*	IncL	80,791
CP-2	*Citrobacter portucalensis*	JAVTRW000000000	5.37	5,19	51.52	6	*bla*CMY-49, *bla*OXA-48, *bla*CTX-M-36*, *bla*CTX-M-36*, *bla*OXA-48, *bla*CTX-M-36*	qnrB1, aph(6)-Id, aph(3'')-Ib, aac(3)-IVa*, aph(4)-Ia	IncL	65,788
CP-3	*Citrobacter portucalensis*	JAVTRX000000000	5.63	5,00	52.04	4	*bla*CTX-M-15, *bla*CTX-M-15, *bla*CMY-129*, *bla*OXA-48	qnrB2, aac(6')-Ib-cr*, arr-3, dfrA27, aadA16, sul1, mph(A)*, tet(A), aph(6)-Id, aph(3'')-Ib*, sul2, sul1, aadA2, dfrA12	IncL	63,024

References

1. Hans JB, Pfennigwerth N, Neumann B, Pfeifer Y, Fischer MA, Eisfeld J, et al. Molecular surveillance reveals the emergence and dissemination of NDM-5-producing Escherichia coli high-risk clones in Germany, 2013 to 2019. Euro Surveill Bull Eur Sur Mal Transm Eur Commun Dis Bull. 2023 Mar;28(10):2200509.

2. Pfennigwerth N, Gatermann SG, Körber-Irrgang B, Hönings R. Phenotypic Detection and Differentiation of Carbapenemase Classes Including OXA-48-Like Enzymes in *Enterobacterales* and *Pseudomonas aeruginosa* by a Highly Specialized Micronaut-S Microdilution Assay. Ledeboer NA, editor. J Clin Microbiol. 2020 Oct 21;58(11):e00171-20.

3. Bolger AM, Lohse M, Usadel B. Trimmomatic: A flexible trimmer for Illumina sequence data. Bioinformatics. 2014 Aug 1;30(15):2114–20.

4. De Coster W, D'Hert S, Schultz DT, Cruts M, Van Broeckhoven C. NanoPack: Visualizing and processing long-read sequencing data. Bioinformatics. 2018;34(15):2666–9.

5. Jolley KA, Bray JE, Maiden MCJ. Open-access bacterial population genomics: BIGSdb software, the PubMLST.org website and their applications. Wellcome Open Res. 2018;3.

6. Carattoli A, Zankari E, Garciá-Fernández A, Larsen MV, Lund O, Villa L, et al. In Silico detection and typing of plasmids using plasmidfinder and plasmid multilocus sequence typing. Antimicrob Agents Chemother. 2014;58(7):3895–903.

7. Feldgarden M, Brover V, Haft DH, Prasad AB, Slotta DJ, Tolstoy I, et al. Validating the AMRFINder tool and resistance gene database by using antimicrobial resistance genotype-phenotype correlations in a collection of isolates. Antimicrob Agents Chemother. 2019;63(11):e00483-19.

8. Siguier P, Perochon J, Lestrade L, Mahillon J, Chandler M. ISfinder: the reference centre for bacterial insertion sequences. Nucleic Acids Res. 2006 Jan 1;34:D32-6.

9. Page AJ, Taylor B, Delaney AJ, Soares J, Seemann T, Keane JA, et al. SNP-sites: rapid efficient extraction of SNPs from multi-FASTA alignments. Microb Genomics.2016;2(4):e000056.

10. Sattler J, Tsvetkov T, Stelzer Y, Schäfer S, Sommer J, Noster J, et al. Emergence of Tn *1999.7*, a New Transposon in *bla* $_{OXA-48}$ -Harboring Plasmids Associated with Increased Plasmid Stability. Antimicrob Agents Chemother. 2022 Nov 15;66(11):e00787-22.

11. Zhou Z, Alikhan NF, Sergeant MJ, Luhmann N, Vaz C, Francisco AP, et al. GrapeTree: visualization of core genomic relationships among 100,000 bacterial pathogens. Genome Res. 2018 Jan 9;28(9):1395–404.

12. Argimón S, Abudahab K, Goater RJE, Fedosejev A, Bhai J, Glasner C, et al. Microreact: visualizing and sharing data for genomic epidemiology and phylogeography. Microb Genomics. 2016 Nov 30;2(11):e000093.

13. Qin S, Cheng J, Wang P, Feng X, Liu HM. Early emergence of OXA-181-producing Escherichia coli ST410 in China. J Glob Antimicrob Resist. 2018;15:215–8.

14. Moussa J, Panossian B, Nassour E, Salloum T, Abboud E, Tokajian S. Detailed characterization of an IncFII plasmid carrying blaOXA-48 from Lebanon. J Antimicrob Chemother. 2020;75(9):2462–5.

15. AbuOun M, Jones H, Stubberfield E, Gilson D, Shaw LP, Hubbard ATM, et al. A genomic epidemiological study shows that prevalence of antimicrobial resistance in Enterobacterales is associated with the livestock host, as well as antimicrobial usage. Microb Genomics. 2021 Oct 5;7(10):000630.
